# Neural Representations of Neuropsychiatric Symptoms in Alzheimer's Disease Continuum Using Pathology‐Based Functional Connectivity Analysis

**DOI:** 10.1002/brb3.70774

**Published:** 2025-09-01

**Authors:** Taein Lee, Yong Jeong, for the Alzheimer's Disease Neuroimaging Initiative

**Affiliations:** ^1^ Department of Bio and Brain Engineering Korea Advanced Institute of Science and Technology Daejeon Republic of Korea

**Keywords:** Alzheimer's disease, functional connectivity, neuropsychiatric symptoms, tau

## Abstract

**Objectives**: Neuropsychiatric symptoms (NPS) in the Alzheimer's disease continuum affect the quality of life for patients and caregivers. Therefore, elucidating the mechanisms of NPS is needed to better understand NPS and enhance patient care. Several studies have investigated them using neuroimaging; however, none have considered the regional coexistence of amyloid‐beta and tau pathologies and its association with functional networks. In this study, we aim to identify the neural correlates of NPS considering the molecular and functional levels of neural representation.

**Methods**: This study included data from amyloid‐positive participants in the Alzheimer's Disease Neuroimaging Initiative database with positron emission tomography (PET), functional magnetic resonance imaging, NPS scores, and demographic data within one year and categorized them into groups with or without NPS factors. NPS were assessed using the neuropsychiatric inventory (NPI) and grouped into affective, apathy, hyperactivity, or psychosis factors. Amyloid‐beta and tau accumulation measured by PET were compared between groups per region. Differences in functional connectivity of NPS‐ or NPS‐type‐specific pathologically altered regions were investigated using seed‐to‐voxel analysis. Further, a generalized linear model was constructed using the NPI score for each factor as the dependent variable, with functional connectivity strength, tau, and amyloid‐beta accumulation as predictors.

**Results**: Significant differences were observed in amyloid‐beta and tau accumulation and functional connectivity between groups. The right middle temporal gyrus (rMTG) was associated with the affective factor, and the right parahippocampal and right fusiform gyri were associated with the apathy factor. Moreover, the generalized linear model was able to predict affective factor severity mainly based on the functional connectivity strength between the rMTG and the left supramarginal and angular gyri.

**Conclusions**: We identified the key regions specific to affective and apathy factors based on the areas with coexistence of amyloid‐beta and tau pathologies and the accompanying altered functional connectivity.

## Graphical abstract

We identified an interconnected disruption of functional connectivity and pathological burden contributing to neuropsychiatric symptoms across the Alzheimer's disease continuum. The right middle temporal gyrus (rMTG) emerged as a key region associated with affective symptoms, right fusiform gyrus (rFFG) and right parahippocampal gyrus (rPH) were linked to apathy‐related symptoms. The brain network map (left panel in the graphical abstract) was visualized with the BrainNetViewer (Xia et al. 2013).

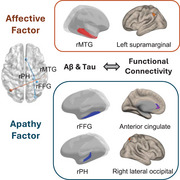

## 1 Introduction

Alzheimer's disease (AD), the most common cause of dementia, presents with a loss of memory, attention, and other cognitive abilities, along with neuropsychiatric symptoms (Kandel et al. 2021). AD is a progressive disease continuum from an asymptomatic phase with AD biomarker evidence (preclinical AD), through mild cognitive impairment (MCI) and/or mild behavioral impairment, to AD dementia (Porsteinsson et al. 2021).

Neuropsychiatric symptoms (NPS) are psychiatric symptoms caused by neurological brain disorders (Miyoshi and Morimura 2010). These are pervasive throughout all AD stages (Eikelboom et al. 2021). The odds of exhibiting behavioral symptoms have been shown to be influenced even during preclinical stages of AD (Ehrenberg et al. 2018). NPS commonly include delusions, hallucinations, agitation, depression, anxiety, euphoria, apathy, disinhibition, irritability, aberrant motor behavior, appetite and eating abnormalities, and nighttime behavior disturbances (Cummings 1997; Cummings et al. 1994). These symptoms are so prevalent that 88.7% of the individuals with amyloid‐positive AD dementia have at least one NPS (Eikelboom et al. 2021). The NPS are also associated with accelerated progression and premature mortality (Peters et al. 2015). Furthermore, they have a largely negative effect on the quality of life for both patients and their caregivers (Shin et al. 2005). Therefore, elucidating the mechanisms underlying the NPS in the AD continuum is necessary for a better understanding of the NPS and improving patient care.

NPS have often been investigated as groups of individual symptoms. Grouping behavioral symptoms may better reflect underlying biological mechanisms and psychological processes than a unitary concept (Robert et al. 2005; Aalten et al. 2007). Examination of NPS subgroups may offer valuable insights for developing treatments that target shared mechanisms (Herrmann et al. 2005; Gauthier et al. 2005). Given these advantages, the present study adopted a factor‐based approach to better capture the neural correlates of NPS.

For examining NPS, prior studies have investigated the neural representation of the NPS using various neuroimaging modalities (Balthazar et al. 2013; Bensamoun et al. 2015; Dang et al. 2022; Krell‐Roesch et al. 2019; Li et al. 2022; Serra et al. 2020; Tissot et al. 2021; Tommasi et al. 2021; Yasuno et al. 2020). Previous studies using positron emission tomography (PET) have reported that AD pathologies, including amyloid‐beta or tau deposition, are related to the NPS. Some studies have suggested that amyloid‐beta deposition is related to the NPS (Bensamoun et al. 2015; Krell‐Roesch et al. 2019), whereas others have reported that tau deposition is more important (Dang et al. 2022; Tissot et al. 2021; Yasuno et al. 2020). However, as tau propagation has been suggested to be influenced by amyloid‐beta deposition and ultimately disrupt cognitive function in AD (He et al. 2017; Lee et al. 2022), coexistence and the interaction of these two pathologies could be related to NPS. A previous study examined the interaction between regional tau pathology and cortical amyloid‐beta in gray matter in relation to NPS (Tommasi et al., 2021); however, the local interaction of amyloid‐beta and tau within the same region has received less attention as a potential NPS‐associated factor. Therefore, the brain regions affected by the coexistence of amyloid‐beta and tau would clarify the intervening mechanism underlying the NPS.

Although PET studies can reveal the underlying pathological mechanisms of the NPS, they focus only on a single brain area. To overcome this limitation, functional brain network models are considered useful for understanding psychopathology (Yuen et al. 2014b). Functional connectivity (FC) has a critical role in explaining NPS across the neurocognitive disorder spectrum, such as Parkinson's disease or Lewy body dementia (Zhao et al. 2024; Rashidi‐Ranjbar et al. 2025; Wright et al. 2025). Especially in AD, altered FC in various brain subnetworks is regarded as a key factor in the pathophysiological mechanisms underlying psychological and behavioral symptoms (Balthazar et al. 2013; Qian et al. 2019; Chang et al. 2020; Serra et al. 2020). For example, changes in the connectivity within the anterior salience network predict behavioral symptoms in patients with AD (Balthazar et al. 2013). Decreased connectivity between the left inferior parietal lobule and the rest of the default mode network is related to delusions in AD (Qian et al. 2019). However, a functional network study necessitates an AD pathology‐based explanation (Serra et al. 2020).

Owing to the limited scope of each modality, multimodal imaging studies have been designed to complement one another when investigating the impact of neuropathology on clinical features (Li et al. 2022). A multimodal imaging study including amyloid‐beta PET, tau PET, blood‐oxygen‐level‐dependent (BOLD) signal, and gray matter volume revealed the link between the NPS and various brain alterations in AD (Li et al. 2022). However, the study focused only on voxel‐based clusters without examining their interaction with other areas.

Here, we combined amyloid‐beta and tau PET imaging with FC derived from resting‐state functional magnetic resonance imaging (rsfMRI) to examine the underlying mechanisms of the NPS based on the coexistence of AD pathologies and its corresponding functional representations. Thus, we aimed to observe the neural representation of the NPS from the molecular to the functional level on the whole‐brain scale, and ultimately identify the key regions specific to each NPS factor and its severity.

## 2 Materials and Methods

### 2.1 Data

Data were obtained from the Alzheimer's Disease Neuroimaging Initiative (ADNI) database (adni.loni.usc.edu). The ADNI was launched in 2003 as a public‐private partnership, led by Principal Investigator Michael W. Weiner, MD. The primary goal of ADNI has been to test whether serial magnetic resonance imaging (MRI), PET, other biological markers, and clinical and neuropsychological assessment can be combined to measure the progression of MCI and early AD.

This study included participants from ADNI with florbetaben (FBB) PET, AV‐1451 PET, structural T1, rsfMRI, NPS scores, Mini‐Mental State Examination (MMSE) (Folstein et al. 1975), Rey Auditory Verbal Learning Test (RAVLT) (Rey 1958), and demographic data at baseline. All data were collected within 1 year. AV‐1451 PET images were selected based on UCBERKELEYAV1451_04_26_22. Since amyloid‐beta is a biomarker of Alzheimer's continuum (Jack et al. 2018), this study included participants whose summary standardized value ratio (SUVR) obtained from FBB PET was >1.08 according to UCBERKELEYFBB_04_26_22, which is the standard of amyloid‐positivity (Landau et al. 2021; Royse et al. 2021). Participants were also diagnosed as cognitively normal (CN), early MCI, late MCI, or AD. The inclusion and exclusion criteria for each diagnosis were based on the ADNI3 clinical protocol (https://adni.loni.usc.edu/methods/documents/).

### 2.2 Assessment of the Neuropsychiatric Symptoms

The NPS were assessed using the neuropsychiatric inventory (NPI). The NPI has 12 items for assessment of the severity, frequency, and caregiver burden of the NPS in patients with AD and other neurodegenerative disorders based on interviews with caregivers (Cummings 1997; Cummings et al. 1994). The score for each item was obtained by multiplying the frequency rating (one to four) and the severity rating (one to three) (Cummings 1997; Cummings et al. 1994). The total score across all items was used to assess overall NPS: participants with a score of zero were classified as NPS (−), indicating no symptoms, while those with scores above zero were classified as NPS (+).

These were classified based on previous studies into: affective, apathy, hyperactivity, and psychosis factors (Aalten et al. 2007; Tondo et al. 2022; Yasuno et al. 2020). The affective factor included depression and anxiety. The apathy factor included apathy with appetite and eating abnormalities. The hyperactivity factor included agitation, disinhibition, irritability, euphoria, and aberrant motor behavior. The psychosis factor included delusions, hallucinations, and night‐time behavioral disturbances.

The score for each factor was the sum of the scores of the symptoms within the factor. Participants with zero scores on the affective factor were categorized into the group without the affective factor (Aff [−]), while those with non‐zero scores were categorized into the group with the affective factor (Aff [+]). Participants were classified in the same manner as for the apathy (Apa [−]/Apa [+]), hyperactivity (Hyp [−]/Hyp [+]), and psychosis (Psy [−]/Psy [+]) factors.

### 2.3 Imaging Analysis

All neuroimaging data, including T1‐weighted MRI, FBB PET, AV‐1451 PET, and rsfMRI data, were collected according to the ADNI protocol (https://adni.loni.usc.edu/methods/). All region‐based analyses were executed on individual participants’ brain surfaces to minimize the distortion of microstructural changes in the brain, as detected by various neuroimaging modalities, including PET.

The T1‐weighted MR was aligned with the anterior commissure‐posterior commissure line (Yamashita 2016). We then segmented and annotated the T1 images using the FreeSurfer software version 6.0.0 (https://surfer.nmr.mgh.harvard.edu/). Additionally, we obtained high‐resolution segmentation (gtmseg.mgz) for use in partial volume correction.

PETsurfer was used to process the co‐registered and averaged PET images provided by ADNI. The PET images were co‐registered with the T1 image and partially volume‐corrected using the geometric transfer matrix method, with the cerebellum as a reference (Greve et al.^2014^, ^2016^). The Gaussian kernel was applied to images according to their rounded values of resolution. The regional tau SUVR for each participant was then calculated.

For rsfMRI data, we excluded the first 24 volumes from images obtained in advanced Siemens and the first five volumes from images obtained in Siemens, Philips, and GE medical systems using FSL 6.0.5.1. (https://www.fmrib.ox.ac.uk/fsl) to remove unstable scans from the first ≈15 s. Considering the difference in repetition time (TR) between the advanced Siemens (0.607 s) and other scanners (3 s), a different number of initial volumes were excluded accordingly. Using CONN (RRID: SCR_009550) release 22.a and SPM (RRID: SCR_007037) release 12.7771, we processed the functional images (Nieto‐Castanon 2020a; Penny et al. 2011; Whitfeld‐Gabrieli and Nieto‐Castanon 2012). The voxel displacement map (VDM) was calculated using phase and magnitude images only for those from the advanced Siemens. The VDM was then used for further field map correction.

The T1 and rsfMRI scans were preprocessed using the CONN pipeline (Nieto‐Castanon 2020b). All the rsfMRI data were realigned, except those from the advanced Siemens scanners, which were realigned using field maps for susceptibility distortion correction (Andersson et al. 2001; Friston et al. 1995). They were then corrected using the SPM slice timing correction (Henson et al. 1999; Sladky et al. 2011). Potential outlier scans were identified using artifact detection tools (https://www.nitrc.org/projects/artifact_detect/) as acquisitions with framewise displacement above 0.9 mm or global BOLD signal changes above five standard deviations (Power et al. 2014; Nieto‐Castanon 2022). The rsfMRI data were co‐registered to T1 (Ashburner and Friston 1997; Studholme et al. 1998) and resampled at the cortical surface (Fischl 2012). Finally, surface‐level rsfMRI data were smoothed using 40 iterative diffusion steps, with approximately an 8 mm FWHM smoothing kernel within the cortical surface (Hagler et al. 2006).

The rsfMRI data were denoised using a standard denoising pipeline including the regression of potential confounding effects, followed by bandpass frequency filtering of the BOLD time‐series between 0.008 and 0.09 Hz (Hallquist et al. 2013). Seed‐to‐voxel FC strength was represented by Fisher‐transformed bivariate correlation coefficients from a weighted general linear model.

### 2.4 Statistical Analysis

CONN, SPSS 27, Python (version 3.6.5), R (version 4.2.3), and ggseg (Mowinckel and Vidal‐Piñeiro 2020) were used for statistical analysis and visualization. Sex and diagnosis were compared between the groups using the chi‐square test or Fisher's exact test. Continuous variables for clinical information were compared between the groups using the independent samples *t*‐test, Welch's *t*‐test, or Mann–Whitney *U* test. Pathologically damaged regions related to overall or specific NPS factors were identified as overlapping regions of increased tau and amyloid‐beta accumulation in the NPS (+) or NPS factor (+) groups. Anatomical labels for the regions were derived from the Desikan–Killany–Tourville (DKT) atlas (Klein and Tourville 2012).

To identify the altered functional connectivity of amyloid‐ and tau‐accumulated regions in relation to the NPS factors, we compared FC of the pathologically disturbed regions with the rest of the brain using seed‐to‐voxel analysis. A seed refers to a brain region labeled in the DKT atlas. In this study, a seed region was defined by significant coaccumulation of both amyloid‐beta and tau between the groups. Voxels that exhibited significantly different connectivity with each seed constituted the cluster. Statistical significance of the connectivity was assessed at the voxel level with an uncorrected *p* < 0.001 and at the cluster level with a family‐wise error (FWE) corrected *p* < 0.05. Computation was performed for 4000 simulations, with age, sex, MMSE score, and education level as covariates. The FSL Harvard–Oxford atlas included in the CONN was used for locating resulting clusters in detail (Frazier et al. 2005; Desikan et al. 2006; Makris et al. 2006; Goldstein et al. 2007).

Through this examination, we identified key regions characterized by pathological and functional changes specifically associated with overall NPS and each NPS factor. Additionally, a generalized linear model was constructed to identify whether the pathological burden and FC of the key regions could explain the severity of symptoms in the NPS (+) and factor‐specific (+) groups. The model included the factor score as a dependent variable, and the SUVR of amyloid‐beta and tau in the seed regions, their interaction, FC between the seed and cluster, age, sex, MMSE, and education as independent variables.

To examine whether the observed neural correlates of NPS or NPS factors were independent of disease progression, additional analyses were conducted within the MCI and AD groups separately. The same statistical analyses were applied to each group. By comparing the resulting pathological and FC patterns with those observed across the AD continuum, this study aimed to explore the relationship between NPS expression and clinical disease stage.

## 3 Results

### 3.1 Demographics and Clinical Characteristics

Overall, 74 participants were included in this study. The demographics of the groups with and without any NPS (NPS [+] and NPS [−]) are summarized in Table 1. Table 2 presents the demographics for each NPS factor. No difference was observed in age, education years, or sex between NPS (+) and NPS (−), and between the positive and negative groups for each factor. As expected, MMSE scores and the ratio of diagnoses differed between all groups except the psychosis factor. Immediate memory performance was also significantly different across groups defined by overall NPS, the affective, apathy, and hyperactivity factors. Furthermore, potential comorbidities were observed between the NPS factors as significant differences in factors other than the target factor across the groups. However, the target factor was the most significantly different between the groups, indicating its main role in group differentiation (Table S1).

**TABLE 1:** Comparison of demographics between groups with and without any neuropsychiatric symptoms.

	NPS		
	NPS (−) (*n* = 28)	NPS (+) (*n* = 46)	*p*
Male^a^	12 (42.8)	24 (52)	0.437
Age^b^	70.7 (5.7)	74 (7.5)	0.051
MMSE^b^	**28.2 (1.6)**	**25.2 (3.5)**	**<0.001**
Education^b^	15.9 (2.4)	16.2 (2.4)	0.367
Diagnosis^a^			
CN	**7 (25)**	**2 (4.3)**	**<0.001**
MCI	**21 (75)**	**23 (50)**	
AD	**0 (0)**	**21 (45.7)**	
NPS factor^a^			
Affective	**0 (0)**	**2 (3.2)**	**<0.001**
Apathy	**0 (0)**	**2.3 (3.5)**	**<0.001**
Hyperactivity	**0 (0)**	**2.9 (4.7)**	**<0.001**
Psychosis	**0 (0)**	**1.4 (2.6)**	**<0.001**
RAVLT^b^			
Immediate memory	**40.1 (11.6)**	**29.4 (11.7)**	**<0.001**
Learning	**5.2 (3)**	**3.2 (2.1)**	**<0.01**
Forgetting	4.6 (3.6)	5.2 (2.4)	0.077
Percent forgetting	**47.8 (35.7)**	**76.4 (33.2)**	**<0.001**

Abbreviations: AD, Alzheimer's disease; CN, cognitive normal; MCI, mild cognitive impairment; MMSE, Mini‐Mental State Examination; NPS (−), group without any neuropsychiatric symptom; NPS (+), group with at least one NPS; NPS, neuropsychiatric symptom; RAVLT, Rey's Auditory Verbal Learning Test.

^a^ Data are presented as numbers (*n*) with corresponding percentages (%);

^b^ Data are presented as mean (standard deviation).

**TABLE 2:** Comparison of demographics between groups with and without neuropsychiatric factor.

	Affective factor			Apathy factor			Hyperactivity factor			Psychosis factor		
	Aff (−) (*n* = 49)	Aff (+) (*n* = 25)	p	Apa (−) (*n* = 51)	Apa (+) (*n* = 23)	p	Hyp (−) (*n* = 50)	Hyp (+) (*n* = 24)	p	Psy(−) (*n* = 58)	Psy(+) (*n* = 16)	*p*
Male^a^	24 (49.0)	12 (48.0)	0.936	22 (43.1)	14 (60.9)	0.158	21 (42.0)	15 (62.5)	0.099	31 (53.4)	5 (31.3)	0.116
Age^b^	72.9 (7.2)	72.5 (6.9)	0.821	72.8 (7.1)	72.7 (7.0)	0.969	71.7 (6.3)	74.9 (8.1)	0.065	72.8 (6.8)	72.5 (8.0)	0.843
MMSE^b^	**27.2** **(2.6)**	**24.7** **(3.7)**	**<0.01**	**27.3** **(2.6)**	**24.3** **(3.6)**	**<0.001**	**26.9** **(3.1)**	**25.3** **(3.4)**	**<0.05**	26.3 (3.3)	26.6 (3.0)	0.458
Education^b^	16.2 (2.5)	15.8 (2.4)	0.237	16.3 (2.3)	15.7 (2.8)	0.184	16.1 (2.4)	16.1 (2.7)	0.488	15.9 (2.6)	16.8 (2.0)	0.134
Diagnosis^a^												
CN	**9** **(18.4)**	**0.0** **(0.0)**	**<0.01**	**8** **(15.7)**	**1** **(4.3)**	**<0.01**	**8** **(16.0)**	**1** **(4.2)**	**<0.05**	8 (13.8)	1 (6.3)	0.273
MCI	**32** **(65.3)**	**12** **(48.0)**		**34** **(66.7)**	**10** **(43.5)**		**32** **(64.0)**	**12** **(50.0)**		36 (62.1)	8 (50.0)	
AD	**8** **(16.3)**	**13** **(52.0)**		**9** **(17.6)**	**12** **(52.2)**		**10** **(20.0)**	**11** **(45.8)**		14 (24.1)	7 (43.8)	
NPS factor^a^												
Affective	**0.0** **(0.0)**	**3.7** **(3.6)**	**<0.001**	**0.9** **(2.7)**	**1.9** **(2.8)**	**<0.05**	**0.6** **(1.4)**	**2.5** **(4.1)**	**<0.05**	**0.9** **(2.0)**	**2.4 (4.4)**	**<0.05**
Apathy	1.2 (2.7)	2.0 (3.4)	0.058	**0.0** **(0.0)**	**4.7** **(3.6)**	**<0.001**	**0.5** **(1.6)**	**3.4** **(4.0)**	**<0.001**	1.5 (3.1)	1.3 (2.2)	0.477
Hyperactivity	**1.1** **(2.6)**	**3.3** **(5.5)**	**<0.05**	**0.6** **(2.1)**	**4.4** **(5.6)**	**<0.001**	**0.0** **(0.0)**	**5.6** **(5.3)**	**<0.001**	1.8 (4.1)	1.9 (3.5)	0.323
Psychosis	**0.4** **(1.2)**	**1.7** **(3.1)**	**<0.05**	0.7 (2.0)	1.1 (2.4)	0.391	0.8 (2.0)	1.1 (2.3)	0.295	**0.0** **(0.0)**	**4.0 (2.9)**	**<0.001**
RAVLT^b^												
Immediate memory	**36.9** **(13)**	**26.8 (9.1)**	**<0.001**	**35.9 (11.9)**	**28.2 (13.1)**	**<0.01**	**35.3 (13.4)**	**29.7 (10.4)**	**<0.05**	33.2 (12.8)	34.4 (12.8)	0.754
Learning	**4.5** **(2.7)**	**2.9** **(2.2)**	**<0.05**	**4.5** **(2.8)**	**2.9** **(2)**	**<0.05**	4.3 (2.9)	3.3 (2)	0.16	**3.9** **(2.8)**	**4.3** **(2.2)**	**0.623**
Forgetting	4.7 (3.3)	5.4 (2.1)	0.333	5.2 (3)	4.4 (2.7)	0.145	5 (3.1)	4.8 (2.6)	0.378	**4.6** **(3.1)**	**6** **(2.2)**	**<0.05**
Percent forgetting	**56.8 (38.6)**	**82.8 (25.6)**	**<0.01**	62.7 (35.1)	72.1 (40.1)	0.081	63.4 (36.1)	70.2 (38.4)	0.142	62.9 (38.2)	75.2 (29.6)	0.171

Abbreviations: AD, Alzheimer's disease; Aff (−), group without the affective factor; Aff (+), group with the affective factor; Apa (−), group without the apathy factor; Apa (+), group with the apathy factor; CN, cognitive normal; Hyp (−), group without the hyperactivity factor; Hyp (+), group with the hyperactivity factor; MCI, mild cognitive impairment; MMSE, Mini‐Mental State Examination; NPS, neuropsychiatric symptoms; Psy (−), group without the psychosis factor; Psy (+), group with the psychosis factor; RAVLT, Rey's Auditory Verbal Learning Test.

^a^ Data are presented as number *(n)* with corresponding percentage (%).

^b^ Data are presented as mean (standard deviation).

### 3.2 Amyloid‐Beta and Tau Accumulation Patterns Related to Overall NPS and Each Factor

**FIGURE 1:**
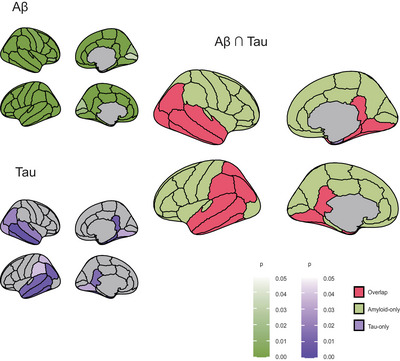
Regions with more accumulation of both tau and Aβ in the group with any neuropsychiatric symptom. Regions with more amyloid‐beta accumulation (green), with more tau accumulation (purple), and overlapping regions where both amyloid‐beta and tau accumulated more (red) in NPS (+).

**FIGURE 2:**
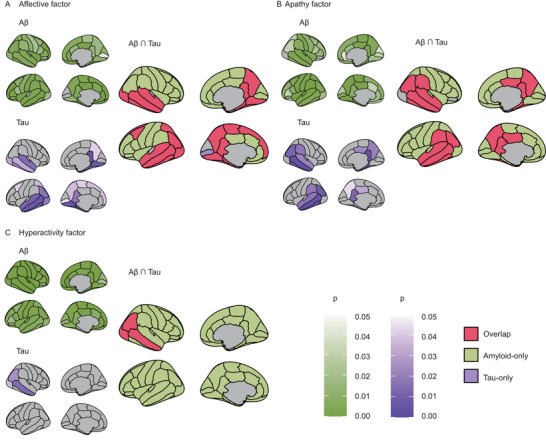
Regions with more accumulation of both tau and Aβ in the group with neuropsychiatric factors. Regions with more amyloid‐beta accumulation (green), with more tau accumulation (purple), and overlapping regions where both amyloid‐beta and tau accumulated more (red) in (A) Aff (+), in (B) Apa (+), and in (C) Hyp (+). Note that there was no significantly different region in tau and amyloid‐beta accumulation for the psychosis factor.

For the NPS (+) group, we identified regions with elevated amyloid‐beta (green) or tau (purple) accumulation and determined overlapping areas exhibiting both pathologies (red), as shown in Figure 1 and Table S2. Greater accumulation in the NPS (+) was observed in 19 regions—including bilateral temporal and parietal cortices such as the fusiform gyrus, inferior and middle temporal gyri—compared to that of NPS (−). We also examined regions with greater pathological burden associated with each NPS factor (Figure 2). Aff (+) had 23 overlapping regions along the temporal lobe to the frontal cortical regions (Figure 2A, Table S3). Apa (+) had a greater accumulation in 20 regions, including the temporal lobe and posterior cingulate, in both hemispheres than that of Apa (−) (Figure 2B, Table S4). Hyp (+) had a greater accumulation only in the right inferior parietal, lateral occipital, and middle temporal gyri than that of Hyp (−) (Figure 2C, Table S5). Moreover, Hyp (+) had less tau accumulation than that of Hyp (−) in bilateral caudal anterior cingulate cortices (Table S5B). No significant difference was observed in the accumulation of both amyloid‐beta and tau between Psy (+) and Psy (−).

### 3.3 NPS‐Specific and Factor‐Specific Regions With AD Pathology Accumulation and FC Changes

To examine whether NPS were associated with changes in FC of pathologically burdened regions, FC was compared between groups using seed regions with elevated amyloid‐beta and tau accumulation. As a result, no significant differences were observed between NPS (+) and NPS (−).

**FIGURE 3:**
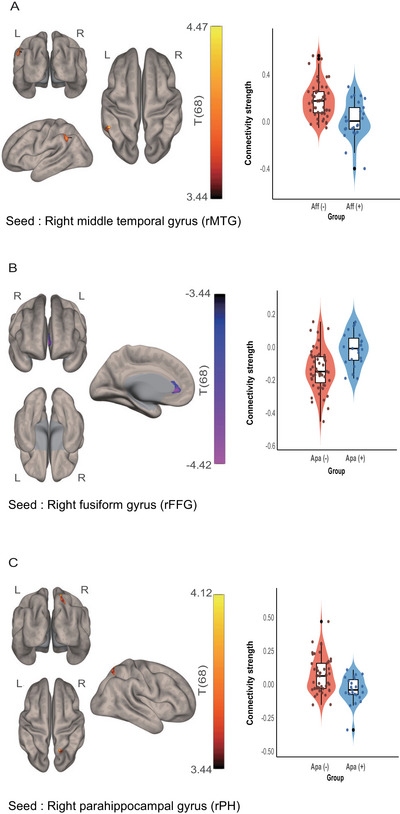
Different functional connectivity of regions with accumulated amyloid‐beta and tau between neuropsychiatric factor (+) and (−) groups. (A) Left: A cluster in the left posterior supramarginal and angular gyri showed different FC with rMTG when contrasting Aff (+) from Aff (−). The rMTG, a seed region, was selected among the regions with higher amyloid‐beta and tau accumulation (Figure 2A (red)) based on the presence of significant functional connectivity differences. Right: Distribution of individual functional connectivity strength between rPH and the cluster. (B) Left: A cluster in the anterior cingulate gyrus had different FC with the rFFG when contrasting Apa (+) from Apa (−). Right: Distribution of individual functional connectivity strength between rFFG and the cluster. (C) Left: A cluster in the right lateral occipital cortex had different FC with the rPH when contrasting Apa (+) from Apa (−). Right: Distribution of individual functional connectivity strength between rFFG and the cluster. Both seed regions in (B) and (C) were selected among the regions with higher amyloid‐beta and tau accumulation (Figure 2B (red)) based on the presence of significant functional connectivity differences. Dots are z‐transformed individual connectivity strength between the seed and the cluster.

In contrast, comparison between Aff (−) and Aff (+) revealed greater negative connectivity of the right middle temporal gyrus (rMTG) with a cluster in the left posterior supramarginal and angular gyri in Aff (+) than in Aff (−) (Effect size: Aff (−), 0.0662 ± 0.4186; Aff (+), −0.1196 ± 0.4061) (Figure 3A, Table 3).

**TABLE 3:** Significant clusters from comparisons of seed‐to‐voxel functional connectivity between groups with and without neuropsychiatric factor.

Seed	Cluster	Cluster size (voxels)	Peak MNI coordinate (x, y, z)	size p‐FWE
**Affective factor**				
rMTG	Left supramarginal gyrus, posterior division; Left angular gyrus; Left lateral occipital cortex, superior division	614	−52 − 45 + 52	0.01275
**Apathy factor**				
rFFG	Cingulate gyrus, anterior division; Left paracingulate gyrus; Subcallosal cortex	355	−04 + 37 − 01	0.04875
rPH	Right lateral occipital cortex, superior division; Right superior parietal lobule	329	+18 − 60 +67	0.0455

*Note*: There was no significant functional connectivity difference for the hyperactivity factor.

Abbreviations: FWE, family‐wise error; rFFG, right fusiform gyrus; rMTG, right middle temporal gyrus; rPH, right parahippocampal gyrus.

For the apathy factor, the right fusiform (rFFG) and the right parahippocampal gyri (rPH) had different FC between Apa (−) and Apa (+). The rFFG had negative connectivity with a cluster in the cingulate gyrus, and the rPH had positive connectivity with a cluster in the right superior lateral occipital cortex. Notably, both the rFFG and rPH had decreased connectivity with clusters in Apa (+) (Effect size for the rFFG: Apa (−), −0.5942 ± 0.3244; Apa (+), −0.4403 ± 0.3104) (Effect size for the rPH: Apa (−), 0.4908 ± 0.3627; Apa (+), 0.3364 ± 0.3470) (Figure 3B, C; Table 3).

For the hyperactivity factor, no FC change was observed in the seed regions (with more amyloid‐beta and tau accumulation) and the bilateral caudal anterior cingulate cortices (which differ from the seed regions, as they had less accumulation in Hyp (+)).

In summary, the rMTG was revealed as the key region of the affective factor, and the rFFG and rPH were the key regions of the apathy factor. Meanwhile, no key regions were identified for the hyperactivity factor.

### 3.4 Predicting the Severity of the Factors With Pathological and Functional Features of the Key Regions

**FIGURE 4:**
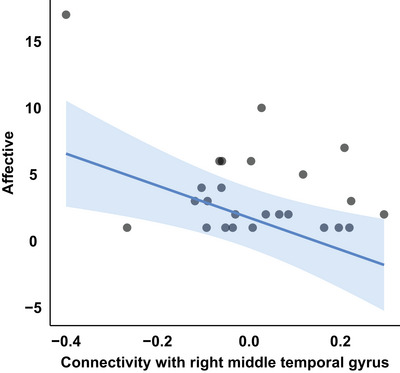
Functional connectivity of rMTG predicting the severity of the affective factor. A stronger negative functional connectivity between the right middle temporal gyrus (rMTG) and left supramarginal/angular gyri was associated with greater severity of affective symptoms. Each dot represents an individual participant's connectivity strength between rMTG and the cluster.

To investigate the relationship between the key regions and the severity of the factors, a generalized linear model was used. A model with rMTG explained the affective factor significantly (adjusted *R*‐squared, 0.3685; *p*, 0.04045). We observed that the FC strength of the rMTG could predict the severity of the affective factor (*p*, 0.00793). As the connectivity between the rMTG and left supramarginal/angular gyri became more negative, the severity of the affective factor increased (Table 4; Figure 4). Although MMSE and the interaction of tau and amyloid‐beta were not statistically significant, they explained the severity of the affective factor marginally (*p* for MMSE, 0.05298; *p* for the interaction term, 0.07476).

**TABLE 4:** Generalized linear model to predict the severity of the affective factor.

Variables	β	Standard error	p‐Value
Intercept	12.39952	15.28838	0.42924
Age	0.09905	0.0992	0.33293
Sex	2.82998	1.64348	0.10435
Education	0.2514	0.33544	0.46446
MMSE	−0.42227	0.20208	0.05298
Connectivity with rMTG	−12.01779	3.96349	0.00793**
Tau SUVR in rMTG	−9.48998	5.54861	0.10652
Amyloid SUVR in rMTG	−10.13096	8.47529	0.24937
Tau: Amyloid SUVR	7.93248	4.16512	0.07476

Abbreviations: MMSE, Mini‐Mental State Examination; rMTG, right middle temporal gyrus; SUVR, standardized uptake value ratio; Tau: Amyloid SUVR, the interaction term of tau and amyloid SUVR in the rMTG; **<0.01.

Meanwhile, the rFFG and rPH could not predict the severity of the apathy factor based on their SUVRs and FC, either separately or together. The model, including only the FFG, showed an adjusted *R*‐squared of 0.11 (*p*, 0.3019) (Table S6). The rPH model was also not significant with an adjusted *R*‐squared of 0.02678 (*p*, 0.4318) (Table S7). When both regions (rFFG and rPH) were included in the model, the adjusted *R*‐squared was 0.1569 (*p*, 0.3255) (Table S8).

### 3.5 Additional Analysis: Stage‐Specific Examination of NPS‐Related Regions

Although the primary focus of this study was on prevalent NPS across the AD continuum, additional analyses were conducted after stratifying participants by clinical stage. Due to the limited number of CN participants presenting with NPS, subgroup analyses were restricted to the MCI and AD groups.

In this approach, the regions identified as factor‐specific in the main analysis did not consistently explain NPS expression in the specific clinical stage. Few brain regions were found to exhibit both pathological burden and FC alterations related to either overall NPS or specific NPS factors in each stage. For overall NPS and the psychosis factor, no region showed a significant discrepancy in both amyloid‐beta and tau burden in either the MCI or the AD stage (Tables S9 and S13).

In MCI participants, the bilateral isthmus of cingulate gyrus (ICgG) and rPH, along with the left middle and superior temporal gyri, exhibited pathological associations with the apathy factor (Table S11). For the hyperactivity factor in AD participants, the bilateral lateral occipital cortex showed increased pathological burden (Table S12). However, none of these regions showed accompanying FC alterations and thus could not be considered key regions for those factors.

Only the left isthmus of the cingulate gyrus (lICgG) emerged as a stage‐specific neural correlate for the affective factor. In MCI participants with the affective factor, the lICgG and rPH demonstrated differential amyloid‐beta and tau accumulation (Table S10). Furthermore, these participants showed decreased FC between lICgG and both the right frontal pole and right postcentral gyrus (Effect size between lICgG and right frontal pole: Aff (−), 0.2933 ± 0.5657; Aff (+), 0.0382 ± 0.5547) (Effect size between lICgG and right postcentral gyrus: Aff (−), −0.6981 ± 0.4661; Aff (+), −0.4971 ± 0.4570) (Figure S1; Table S14). However, this region did not significantly predict affective severity in MCI participants (adjusted *R*‐squared, ‐0.0037; *p*, 0.5962) (Table S15).

## 4 Discussion

In this study, we elucidated the neural representation of the NPS in the AD continuum using pathology‐based FC analysis. We revealed that the rMTG was the key region specific to the affective factor, while the rFFG and rPH were specific to the apathy factor. This aligns with previous findings suggesting tau in the temporal lobe as a pathology related to the affective and apathy factors (Tissot et al. 2021; Dang et al. 2022; Yasuno et al. 2020). We demonstrated that these regions exhibit the coexistence of amyloid‐beta and tau accumulation along with disrupted functional connectivity, suggesting a convergent mechanism linking molecular pathology to network‐level dysfunction in NPS.

The key regions are known as regions of initial tau accumulation that interact with amyloid‐beta for spreading. Specifically, the FFG and PH—key regions of the apathy factor—are involved in tau‐spreading via interaction with amyloid‐beta in deposited remote regions (Lee et al. 2022). Meanwhile, the MTG, the key region of the affective factor, is topographically adjacent to the inferior temporal gyrus, the tau propagation hub through local amyloid‐beta and tau interactions (Lee et al. 2022). Consequently, the close interaction between tau and amyloid‐beta in tau propagation initiation areas is likely to render these regions highly susceptible to NPS development.

In terms of functional connectivity, the rMTG had increased negative FC with the left supramarginal and angular gyri in the Aff (+) group. A previous study demonstrated that the rMTG was activated in response to threat stimuli, especially those directed toward the individual (Lima Portugal et al. 2020). Conversely, the inferior parietal lobule, including the supramarginal and angular gyri, had decreased functional activation in association with affective instability (Hua et al. 2020). Based on the negative connectivity of these two regions, we speculated that the pathological damage in the rMTG may be related to functional network disruption, causing excessive sensitivity toward self‐directed negative stimuli and impaired emotional regulation, which are, in turn, associated with the affective factor.

In case of the apathy factor, the rFFG had significantly reduced connectivity with the anterior cingulate gyrus. The rFFG participates in emotion perception and generation in response to external stimuli (Morawetz et al. 2020). Furthermore, the pregenual anterior cingulate cortex serves as a hub in an efferent salience network, delivering viscero‐autonomic, emotional, and behavioral responses to salient stimuli detected in the frontoinsula (Zhou and Seeley 2014). Similarly, the rPH and right lateral occipital cortex had decreased connectivity in the Apa (+) group. The rPH processes emotions, particularly passive reactivity (McTeague et al. 2020) and emotional memory (Loos et al. 2019). As the right superior lateral occipital cortex is a part of the visual network (Uddin et al. 2019), our findings suggest that decreased connectivity between perceptual regions and those implicated in motivation or emotion processing may contribute to the apathy factor along the AD continuum.

We were unable to identify the regions associated with the hyperactivity and psychosis factors. Although some regions exhibited differences in pathological damage between Hyp (+) and Hyp (−), these areas did not accompany significant FC changes. The bilateral caudal anterior cingulate cortices with more tau accumulation in Hyp (−) than in Hyp (+) also revealed no significant FC changes on supplementary analyses incorporating all significant brain regions out of our primary aim. Regarding the psychosis factor, there was no region with significantly different accumulation of amyloid‐beta and tau. It is noteworthy that most prior studies have explained the association of tauopathy with symptoms included in the affective and apathy factors, but not the hyperactivity or psychosis factor (Dang et al. 2022; Tommasi et al., 2021; Yasuno et al. 2020). In relation to hyperactivity, AD pathologies appear to be less associated with its manifestation compared to other risk factors such as phosphorylated TDP‐43 (Sennik et al. 2016; Borges et al. 2019). Similarly, psychosis may also arise from alternative mechanisms. For instance, structural (cortical thickness) and pathological abnormalities (tau, α‐synuclein) have been shown to influence psychosis differently in a sex‐dependent manner (Whitehead et al. 2012; Koppel et al. 2014). Therefore, we suggest that another mechanism may underlie both the hyperactivity and psychosis factors.

We further investigated the possibility of the identified key regions predicting the severity of NPS, based on their associated pathology and FC patterns. We employed a model for the affective factor with an interaction term between amyloid‐beta and tau SUVR in the rMTG and its FC strength with a significant cluster. Here, we observed that as the FC between the rMTG and the left supramarginal and angular gyri became more negative, the affective factor worsened. Additionally, MMSE and the interaction between tau and amyloid‐beta marginally explained the severity of the affective factor. Given the well‐established association between cognitive decline and NPS (Lü et al. 2021; Sabates et al. 2023), lower MMSE scores may reflect greater susceptibility to affective symptoms. Furthermore, given that the rMTG is a region where local interaction between amyloid‐beta and tau is highly expected (Lee et al. 2022), it is plausible that increased amyloid‐beta may facilitate tau accumulation, thereby contributing—albeit modestly—to the severity of the affective factor. However, the model using the rFFG or rPH did not significantly explain the severity of the apathy factor. These results suggest a potential difference in their ability to represent NPS. The core region of the affective factor seemed to explain its presence and severity. Conversely, the core regions of the apathy factor seemed to capture only its presence.

The impact of the temporal lobe across various AD symptoms was consolidated through the present study. While the investigation primarily focused on the temporal region as a neural underpinning of NPS, this region has also been widely recognized for its involvement in cognitive malfunction in AD. Alterations in FC of the medial temporal region have been reported to accompany memory reduction and cognitive decline across AD stages (Berron et al. 2020; Dautricourt et al. 2021). Furthermore, tau accumulation in the temporal region has been associated with impairments in episodic memory, semantic memory, and language abilities (Bejanin et al. 2017). By integrating findings from the current study, it can be inferred that a wide range of AD symptoms—encompassing both cognitive and behavioral domains—share a regional disruption specific to the temporal lobe.

In addition to the shared neural correlates observed across different functional domains, interdependence among NPS, cognitive impairment, and AD progression was also verified. Demographic comparisons revealed significantly different MMSE scores, RAVLT performance, and diagnostic group distributions between NPS (factor) (+) and NPS (factor) (−), except in the case of the psychosis factor. Depression, anxiety, and apathy have been reported to be associated with reduced learning capacity and episodic memory performance (Lü et al. 2021). Furthermore, the prevalence of NPS has been shown to increase in relation to disease progression (Chen et al. 2021). These findings indicate that AD is characterized by co‐occurring disruptions in behavior and cognition that are closely interlinked throughout the disease course.

Although the current study was designed to examine NPS across the AD continuum—reflecting the continuous development of NPS throughout disease progression—additional analyses were conducted by stratifying participants into MCI or AD stage to explore potential stage‐specific neural correlates of NPS. This approach, however, yielded minimal evidence of representative brain regions explaining specific NPS factors within a particular disease stage. Only lICgG, characterized by amyloid‐beta and tau aggregates, exhibited significantly decreased functional connectivity with the right frontal pole and postcentral gyrus among participants expressing the affective factor during the MCI stage. Such stage‐specific analyses, while informative, may not fully account for the broader, overlapping mechanisms underlying behavioral and cognitive symptoms throughout the AD continuum.

Moreover, we observed potential co‐occurrences of the NPS factors as significant differences in non‐target NPS factors between the groups. This indicates that some symptoms in each factor could be regarded as aspects of other factors when reported by caregivers. For instance, apathy and depression share some similarities in their clinical presentations (Steffens et al. 2022; Mortby et al. 2022; Yuen et al. 2014a). Furthermore, hyperactivity symptoms, including agitation and aberrant motor behavior, may not be entirely dissociated from incident anxiety, depression, and apathy (Aalten et al. 2007; Liu et al. 2020). Although comorbidities across NPS factors may introduce overlap, we followed widely accepted NPS factor classification and confirmed that the target factor exhibited the most distinct group differences. This supports the interpretation that observed differences in neural correlates are primarily driven by the targeted NPS factor.

This study had some limitations. First, the sample size was relatively small, as we only included participants with all the necessary data. This prevented us from controlling the medication effects that might influence the NPS. The proportion of participants taking antidepressants and other behavioral medications was reported in Table S16. Second, as we tried to extract values at the regional level by using the same atlas across modalities in each participant space, surface‐based analysis with the DKT atlas was applied, resulting in the exclusion of subcortical regions. Since frontal‐limbic circuits have been implicated in multiple NPS, including both affective and apathy factors (Tagariello et al. 2009; Sampath et al. 2017; Wang et al. 2017; Chen et al. 2021), we expect that further analysis incorporating subcortical regions could better elucidate the altered FC between frontal cortical areas and the hippocampus/amygdala, as well as their pathological burden.

## 5 Conclusion

In conclusion, the pathology‐based FC analysis presented in this study offers an integrated framework for understanding the neural representation of the NPS across the AD continuum. By combining pathological and functional information, key regions were identified: rMTG for the affective factor, and rPH and rFFG for the apathy factor. We demonstrated the interconnected disruption of the different systems implicated in NPS through these key regions. These key regions can provide clinically meaningful information. Since these regions represent converging points of both pathological accumulation and network dysfunction, they may serve as targets not only for pathology‐specific pharmacological treatments but also for neuromodulatory interventions such as transcranial magnetic stimulation. In addition, the predictive model of the affective factor suggests potential as a biomarker for monitoring symptom progression. The integration of biologically based predictive models and traditional caregiver reports could provide more objective assessments of patient status. This may enable the earlier detection of symptoms and the development of more tailored interventions. Taken together, this study ultimately contributes to a deeper understanding of NPS pathophysiology and lays the groundwork for functional and pathology‐informed diagnostic and therapeutic strategies, thus providing benefits to patients and their caregivers.

## Author Contributions

**Taein Lee**: investigation, methodology, visualization, writing – original draft, formal analysis. **Yong Jeong**: supervision, funding acquisition, writing – review and editing, conceptualization, project administration, methodology, resources.

## Conflicts of Interest

The authors declare no conflicts of interest.

## Peer Review

The peer review history for this article is available at https://publons.com/publon/10.1002/brb3.70774.

## Supporting information




**Supplementary material**: brb370774‐sup‐0001‐SuppMat.docx

**Supporting figure S1**: brb370774‐sup‐0002‐figureS1.pdf

## Data Availability

The data for this study were obtained from ADNI and are available through its website.
