# Supplementary material for: Neural Representations of Neuropsychiatric Symptoms in Alzheimer's Disease Continuum Using Pathology‐Based Functional Connectivity Analysis
Source: Brain Behav. 2025 Sep 1;15(9):e70774. doi: 10.1002/brb3.70774 (PMC12402403; doi:10.1002/brb3.70774)
Supplement: Supplementary file 1 — Supplementary material: brb370774‐sup‐0001‐SuppMat.docx [file BRB3-15-e70774-s001.docx]

**Supplementary material**

| **A Affective factor** | | | | |
| --- | --- | --- | --- | --- |
|  | **Shapiro test for Aff (+) (p)** | **Shapiro test for Aff (-) (p)** | **Levene test**  **(p)** | **Group comparison (p)** |
| **Age** | 0.895581305 | 0.912503362 | 0.604480592 | 0.821325992 |
| **MMSE** | 0.203974769 | 4.50E-05 | 0.036358127 | 0.001193941 |
| **Education** | 0.087526269 | 0.005842853 | 0.957227199 | 0.237859825 |
| **Affective** | 1.88E-05 | 1 | 3.73E-06 | 4.70E-17 |
| **Apathy** | 2.09E-06 | 2.06E-11 | 0.261968775 | 0.058407152 |
| **Hyperactivity** | 2.68E-06 | 5.88E-12 | 0.02159841 | 0.032190285 |
| **Psychosis** | 1.03E-06 | 1.06E-12 | 0.011975549 | 0.014207519 |
|  |  |  |  |  |
| **B Apathy factor** | | | | |
|  | **Shapiro test for Apa (+) (p)** | **Shapiro test for Apa (-) (p)** | **Levene test**  **(p)** | **Group comparison (p)** |
| **Age** | 0.431981 | 0.611558 | 0.957448 | 0.968985 |
| **MMSE** | 0.300426 | 6.94E-06 | 0.104059 | 0.000261 |
| **Education** | 0.038722 | 0.008191 | 0.195119 | 0.183879 |
| **Affective** | 5.79E-05 | 3.41E-13 | 0.157621 | 0.024768 |
| **Apathy** | 0.007804 | 1 | 1.80E-10 | 3.47E-17 |
| **Hyperactivity** | 0.000264 | 6.99E-14 | 7.64E-05 | 7.28E-06 |
| **Psychosis** | 2.27E-07 | 7.97E-13 | 0.472092 | 0.391317 |
|  |  |  |  |  |
| **C Hyperactivity factor** | | | | |
|  | **Shapiro test for Hyp (+) (p)** | **Shapiro test for Hyp (-) (p)** | **Levene test**  **(p)** | **Group comparison (p)** |
| **Age** | 0.653697 | 0.847338 | 0.182535 | 0.064868 |
| **MMSE** | 0.100901 | 2.25E-06 | 0.337312 | 0.010723 |
| **Education** | 0.056416 | 0.008104 | 0.541585 | 0.488316 |
| **Affective** | 4.82E-06 | 2.49E-11 | 0.005258 | 0.022933 |
| **Apathy** | 0.000444 | 3.40E-13 | 4.02E-06 | 1.35E-05 |
| **Hyperactivity** | 0.000722 | 1 | 1.17E-08 | 4.07E-17 |
| **Psychosis** | 1.56E-07 | 1.12E-12 | 0.541882 | 0.295458 |
|  |  |  |  |  |
| **D Psychosis factor** | | | | |
|  | **Shapiro test for Psy (+) (p)** | **Shapiro test for Psy (-) (p)** | **Levene test**  **(p)** | **Group comparison (p)** |
| **Age** | 0.524124 | 0.781815 | 0.997879 | 0.84313 |
| **MMSE** | 0.093835 | 3.76E-05 | 0.98653 | 0.457813 |
| **Education** | 0.125675 | 0.001576 | 0.288237 | 0.134388 |
| **Affective** | 2.04E-05 | 3.31E-12 | 0.054422 | 0.022058 |
| **Apathy** | 4.54E-05 | 4.54E-12 | 0.823296 | 0.477648 |
| **Hyperactivity** | 3.07E-05 | 1.10E-12 | 0.886268 | 0.323413 |
| **Psychosis** | 0.00224 | 1 | 5.12E-08 | 1.37E-17 |

Supplementary Table 1. Detailed statistical results for demographics In all group comparisons, the target factor has the lowest p-value between groups (yellow part).

Abbreviations: Aff (-), group without the affective factor; Aff (+), group with the affective factor; Apa (-), group without the apathy factor; Apa (+), group with the apathy factor; Hyp (-), group without the hyperactivity factor; Hyp (+), group with the hyperactivity factor; Psy (-), group without the psychosis factor; Psy (+), group with the psychosis factor; MMSE, Mini-Mental State Examination

* When p-values of Shapiro test in each group were both not significant(>= 0.05) and the result of Levene test was also not significant (>=0.05), group comparison was conducted with independent samples t-test.

* When p-values of Shapiro test in each group were both not significant(>= 0.05) and the result of Levene test was significant (<0.05), group comparison was conducted with Welch’s t-test

* When one or both of p-values of Shapiro test in each group was significant(< 0.05), group comparison was conducted with Mann-Whitney U test

**A Aβ**

| **Region** | **NPS (-)** | | **NPS (+)** | | **p-value** |
| --- | --- | --- | --- | --- | --- |
|  | **Mean (SD)** | **Median (IQR)** | **Mean (SD)** | **Median (IQR)** |  |
| Left caudalanteriorcingulate | 1.1 (0.3) | 1.1 (0.8-1.4) | 1.4 (0.4) | 1.5 (1.2-1.6) | 0.000428 |
| Left caudalmiddlefrontal | 1.0 (0.4) | 1.0 (0.7-1.2) | 1.4 (0.3) | 1.4 (1.1-1.6) | 0.000229 |
| Left cuneus | 0.8 (0.4) | 0.7 (0.6-0.9) | 0.9 (0.3) | 0.8 (0.7-1.1) | 0.023018 |
| Left entorhinal | 0.6 (0.1) | 0.6 (0.5-0.7) | 0.7 (0.2) | 0.6 (0.6-0.8) | 0.034197 |
| Left fusiform | 0.9 (0.3) | 0.8 (0.7-1.0) | 1.2 (0.3) | 1.1 (1.0-1.4) | 6.44E-05 |
| Left inferiorparietal | 1.0 (0.4) | 0.9 (0.8-1.3) | 1.4 (0.3) | 1.4 (1.2-1.7) | 4.90E-05 |
| Left inferiortemporal | 1.0 (0.3) | 0.9 (0.7-1.2) | 1.3 (0.3) | 1.3 (1.1-1.6) | 7.05E-05 |
| Left isthmuscingulate | 1.0 (0.3) | 1.0 (0.8-1.2) | 1.3 (0.3) | 1.2 (1.1-1.4) | 0.00069 |
| Left lateraloccipital | 0.9 (0.4) | 0.8 (0.7-1.1) | 1.1 (0.3) | 1.1 (0.9-1.2) | 0.001878 |
| Left lateralorbitofrontal | 1.1 (0.3) | 1.0 (0.9-1.3) | 1.4 (0.4) | 1.4 (1.2-1.6) | 0.000639 |
| Left lingual | 0.6 (0.3) | 0.6 (0.5-0.7) | 0.8 (0.3) | 0.7 (0.6-0.9) | 0.001358 |
| Left medialorbitofrontal | 1.0 (0.3) | 1.0 (0.8-1.2) | 1.4 (0.4) | 1.4 (1.1-1.7) | 6.79E-05 |
| Left middletemporal | 1.0 (0.4) | 0.9 (0.7-1.2) | 1.3 (0.3) | 1.4 (1.1-1.6) | 4.27E-05 |
| Left parahippocampal | 0.7 (0.2) | 0.6 (0.5-0.7) | 0.9 (0.2) | 0.9 (0.7-1.0) | 4.79E-05 |
| Left paracentral | 0.9 (0.3) | 0.8 (0.7-1.0) | 1.2 (0.3) | 1.2 (0.9-1.4) | 0.000366 |
| Left parsopercularis | 1.0 (0.4) | 0.9 (0.8-1.2) | 1.3 (0.3) | 1.4 (1.1-1.5) | 0.000378 |
| Left parsorbitalis | 1.1 (0.4) | 1.0 (0.9-1.3) | 1.3 (0.3) | 1.4 (1.1-1.5) | 0.002132 |
| Left parstriangularis | 1.1 (0.4) | 1.0 (0.9-1.4) | 1.4 (0.4) | 1.5 (1.3-1.7) | 0.000443 |
| Left pericalcarine | 0.7 (0.6) | 0.6 (0.4-0.9) | 0.9 (0.5) | 0.8 (0.6-1.2) | 0.014258 |
| Left postcentral | 0.8 (0.3) | 0.7 (0.6-1.0) | 1.1 (0.3) | 1.2 (0.9-1.3) | 3.38E-05 |
| Left posteriorcingulate | 1.1 (0.4) | 1.1 (0.9-1.3) | 1.5 (0.3) | 1.5 (1.3-1.7) | 6.32E-05 |
| Left precentral | 0.8 (0.2) | 0.7 (0.7-0.9) | 1.1 (0.3) | 1.1 (0.8-1.3) | 0.000557 |
| Left precuneus | 1.1 (0.4) | 1.0 (0.8-1.4) | 1.6 (0.3) | 1.6 (1.4-1.7) | 5.57E-06 |
| Left rostralanteriorcingulate | 1.1 (0.3) | 1.0 (0.9-1.3) | 1.4 (0.4) | 1.4 (1.2-1.6) | 0.00019 |
| Left rostralmiddlefrontal | 1.2 (0.4) | 1.1 (0.9-1.5) | 1.6 (0.4) | 1.6 (1.4-1.8) | 2.10E-05 |
| Left superiorfrontal | 1.0 (0.4) | 0.9 (0.8-1.3) | 1.4 (0.4) | 1.4 (1.1-1.7) | 1.79E-05 |
| Left superiorparietal | 1.0 (0.4) | 0.9 (0.7-1.1) | 1.4 (0.3) | 1.4 (1.2-1.6) | 1.26E-05 |
| Left superiortemporal | 0.9 (0.3) | 0.8 (0.6-1.0) | 1.2 (0.3) | 1.1 (0.9-1.4) | 3.59E-05 |
| Left supramarginal | 1.0 (0.3) | 0.9 (0.7-1.1) | 1.4 (0.3) | 1.4 (1.2-1.5) | 2.01E-06 |
| Left transversetemporal | 0.8 (0.5) | 0.7 (0.5-0.9) | 1.2 (0.4) | 1.3 (0.9-1.4) | 0.000163 |
| Left insula | 0.8 (0.2) | 0.8 (0.7-0.9) | 1.1 (0.3) | 1.1 (0.9-1.3) | 9.91E-05 |
| Right caudalanteriorcingulate | 1.1 (0.3) | 1.0 (0.9-1.3) | 1.3 (0.3) | 1.4 (1.2-1.5) | 0.00056 |
| Right caudalmiddlefrontal | 1.0 (0.4) | 0.9 (0.8-1.2) | 1.3 (0.3) | 1.4 (1.1-1.6) | 0.000491 |
| Right cuneus | 0.8 (0.4) | 0.6 (0.6-0.8) | 0.9 (0.3) | 0.8 (0.7-1.0) | 0.019081 |
| Right fusiform | 0.9 (0.3) | 0.8 (0.6-1.0) | 1.1 (0.3) | 1.1 (0.9-1.3) | 0.000253 |
| Right inferiorparietal | 1.1 (0.4) | 1.0 (0.8-1.3) | 1.4 (0.3) | 1.4 (1.2-1.6) | 0.000137 |
| Right inferiortemporal | 1.0 (0.3) | 0.8 (0.7-1.1) | 1.3 (0.3) | 1.3 (1.1-1.5) | 6.30E-05 |
| Right isthmuscingulate | 1.1 (0.3) | 1.0 (0.9-1.3) | 1.3 (0.3) | 1.3 (1.1-1.4) | 0.001587 |
| Right lateraloccipital | 0.9 (0.5) | 0.8 (0.7-1.0) | 1.1 (0.3) | 1.1 (0.9-1.2) | 0.001599 |
| Right lateralorbitofrontal | 1.1 (0.3) | 1.0 (0.9-1.3) | 1.4 (0.3) | 1.4 (1.2-1.6) | 0.000839 |
| Right lingual | 0.6 (0.3) | 0.5 (0.5-0.6) | 0.8 (0.3) | 0.7 (0.6-0.8) | 0.002903 |
| Right medialorbitofrontal | 1.0 (0.3) | 1.0 (0.8-1.3) | 1.4 (0.4) | 1.4 (1.2-1.6) | 2.80E-05 |
| Right middletemporal | 1.0 (0.4) | 0.9 (0.7-1.1) | 1.3 (0.3) | 1.3 (1.2-1.5) | 0.000108 |
| Right parahippocampal | 0.7 (0.2) | 0.7 (0.5-0.8) | 0.9 (0.2) | 0.9 (0.7-1.0) | 6.54E-05 |
| Right paracentral | 0.9 (0.3) | 0.9 (0.8-1.1) | 1.2 (0.4) | 1.3 (0.9-1.4) | 0.001912 |
| Right parsopercularis | 1.1 (0.4) | 1.1 (0.7-1.3) | 1.3 (0.3) | 1.3 (1.2-1.5) | 0.002177 |
| Right parsorbitalis | 1.1 (0.4) | 1.1 (0.8-1.3) | 1.3 (0.3) | 1.3 (1.2-1.5) | 0.011317 |
| Right parstriangularis | 1.1 (0.4) | 1.1 (0.8-1.3) | 1.4 (0.3) | 1.5 (1.3-1.6) | 0.000397 |
| Right pericalcarine | 0.8 (0.6) | 0.6 (0.4-0.9) | 1.0 (0.6) | 1.0 (0.6-1.2) | 0.033368 |
| Right postcentral | 0.8 (0.4) | 0.7 (0.6-1.0) | 1.1 (0.3) | 1.2 (0.9-1.3) | 0.000525 |
| Right posteriorcingulate | 1.1 (0.4) | 1.1 (0.8-1.4) | 1.4 (0.4) | 1.4 (1.2-1.7) | 0.000662 |
| Right precentral | 0.9 (0.3) | 0.8 (0.7-1.0) | 1.0 (0.3) | 1.1 (0.8-1.2) | 0.004194 |
| Right precuneus | 1.1 (0.4) | 1.2 (0.8-1.4) | 1.5 (0.4) | 1.5 (1.3-1.7) | 0.00015 |
| Right rostralanteriorcingulate | 1.1 (0.3) | 1.0 (0.9-1.3) | 1.4 (0.4) | 1.4 (1.2-1.6) | 0.000125 |
| Right rostralmiddlefrontal | 1.2 (0.4) | 1.2 (0.8-1.4) | 1.6 (0.4) | 1.6 (1.4-1.8) | 4.62E-05 |
| Right superiorfrontal | 1.0 (0.4) | 1.0 (0.8-1.2) | 1.4 (0.4) | 1.4 (1.1-1.6) | 0.000145 |
| Right superiorparietal | 1.0 (0.4) | 0.9 (0.6-1.2) | 1.3 (0.3) | 1.3 (1.1-1.5) | 0.000156 |
| Right superiortemporal | 0.8 (0.3) | 0.7 (0.6-1.0) | 1.1 (0.3) | 1.2 (0.9-1.4) | 4.79E-05 |
| Right supramarginal | 1.0 (0.4) | 0.9 (0.7-1.3) | 1.3 (0.3) | 1.4 (1.1-1.4) | 0.000248 |
| Right transversetemporal | 0.8 (0.5) | 0.7 (0.5-0.9) | 1.1 (0.4) | 1.2 (0.8-1.3) | 0.001542 |
| Right insula | 0.8 (0.3) | 0.7 (0.6-1.0) | 1.1 (0.3) | 1.1 (0.9-1.2) | 0.000161 |

**B Tau**

| **Region** | **NPS (-)** | | **NPS (+)** | | **p-value** |
| --- | --- | --- | --- | --- | --- |
|  | **Mean (SD)** | **Median (IQR)** | **Mean (SD)** | **Median (IQR)** |  |
| Left entorhinal | 1.6 (0.6) | 1.3 (1.1-2.0) | 2.0 (0.5) | 2.0 (1.5-2.3) | 0.001749 |
| Left fusiform | 1.6 (0.9) | 1.2 (1.2-1.7) | 1.7 (0.6) | 1.5 (1.3-2.0) | 0.003003 |
| Left inferiorparietal | 1.6 (0.7) | 1.3 (1.2-1.7) | 1.8 (0.9) | 1.6 (1.3-2.0) | 0.010065 |
| Left inferiortemporal | 1.8 (0.8) | 1.4 (1.3-1.9) | 2.0 (0.6) | 1.8 (1.5-2.3) | 0.011491 |
| Left isthmuscingulate | 1.4 (0.6) | 1.1 (1.0-1.5) | 1.5 (0.6) | 1.3 (1.1-1.6) | 0.014665 |
| Left lingual | 1.2 (0.5) | 1.1 (1.0-1.2) | 1.3 (0.5) | 1.1 (1.0-1.3) | 0.031739 |
| Left middletemporal | 1.7 (0.9) | 1.3 (1.2-1.7) | 1.9 (0.7) | 1.6 (1.4-2.1) | 0.008407 |
| Left superiorparietal | 1.3 (0.6) | 1.1 (1.1-1.3) | 1.5 (0.8) | 1.3 (1.1-1.6) | 0.03549 |
| Left superiortemporal | 1.4 (0.5) | 1.2 (1.1-1.4) | 1.4 (0.4) | 1.3 (1.2-1.5) | 0.012004 |
| Left supramarginal | 1.4 (0.5) | 1.2 (1.1-1.5) | 1.5 (0.6) | 1.3 (1.2-1.6) | 0.038183 |
| Right entorhinal | 1.6 (0.6) | 1.4 (1.2-1.8) | 2.0 (0.6) | 2.0 (1.6-2.5) | 0.001029 |
| Right fusiform | 1.7 (0.9) | 1.3 (1.2-1.6) | 1.8 (0.6) | 1.5 (1.3-2.2) | 0.011161 |
| Right inferiorparietal | 1.6 (0.8) | 1.3 (1.2-1.8) | 1.9 (1.0) | 1.5 (1.3-2.1) | 0.021541 |
| Right inferiortemporal | 1.8 (0.9) | 1.5 (1.2-1.8) | 2.0 (0.7) | 1.8 (1.4-2.4) | 0.012718 |
| Right isthmuscingulate | 1.4 (0.7) | 1.1 (1.0-1.4) | 1.5 (0.8) | 1.3 (1.1-1.6) | 0.009483 |
| Right lateraloccipital | 1.7 (1.2) | 1.2 (1.1-1.4) | 1.7 (0.8) | 1.4 (1.2-2.0) | 0.029059 |
| Right lingual | 1.3 (0.7) | 1.0 (1.0-1.1) | 1.3 (0.5) | 1.1 (1.0-1.3) | 0.031742 |
| Right middletemporal | 1.7 (0.9) | 1.3 (1.2-1.7) | 1.9 (0.8) | 1.7 (1.4-2.3) | 0.005098 |
| Right parahippocampal | 1.4 (0.5) | 1.2 (1.1-1.5) | 1.5 (0.4) | 1.5 (1.4-1.7) | 0.006994 |
| Right superiortemporal | 1.4 (0.6) | 1.2 (1.1-1.3) | 1.4 (0.4) | 1.3 (1.1-1.4) | 0.01934 |

**C Aβ and tau**

| **Overlapping regions** | |
| --- | --- |
| Left entorhinal | Right fusiform |
| Left fusiform | Right inferiorparietal |
| Left inferiorparietal | Right inferiortemporal |
| Left inferiortemporal | Right isthmuscingulate |
| Left isthmuscingulate | Right lateraloccipital |
| Left lingual | Right lingual |
| Left middletemporal | Right middletemporal |
| Left superiorparietal | Right parahippocampal |
| Left superiortemporal | Right superiortemporal |
| Left supramarginal |  |

Supplementary Table 2. Regions with significantly different pathology between NPS (+) and NPS (-) (A) Comparison of Aβ, (B) Comparison of tau. (C) Brain regions showing differential Aβ and tau burden, chosen as seed regions for subsequent functional connectivity analysis.

Abbreviations: NPS (-), group without any NPS; NPS (+), group with at least one NPS; SD, standard deviation; IQR, interquartile range

**A Aβ**

| **Region** | **Aff (-)** | | **Aff (+)** | | **p-value** |
| --- | --- | --- | --- | --- | --- |
|  | **Mean (SD)** | **Median (IQR)** | **Mean (SD)** | **Median (IQR)** |  |
| Left caudalanteriorcingulate | 1.2 (0.4) | 1.2 (0.9-1.5) | 1.5 (0.3) | 1.5 (1.2-1.6) | 0.004123 |
| Left caudalmiddlefrontal | 1.2 (0.4) | 1.1 (0.8-1.5) | 1.4 (0.3) | 1.4 (1.2-1.5) | 0.012174 |
| Left fusiform | 1.0 (0.3) | 0.9 (0.7-1.2) | 1.2 (0.3) | 1.1 (1.0-1.3) | 0.009422 |
| Left inferiorparietal | 1.2 (0.4) | 1.2 (0.9-1.6) | 1.4 (0.3) | 1.4 (1.3-1.5) | 0.00632 |
| Left inferiortemporal | 1.1 (0.4) | 1.0 (0.8-1.4) | 1.4 (0.3) | 1.3 (1.2-1.6) | 0.004661 |
| Left isthmuscingulate | 1.1 (0.3) | 1.1 (0.9-1.3) | 1.3 (0.2) | 1.3 (1.2-1.5) | 0.006171 |
| Left lateraloccipital | 1.0 (0.4) | 0.9 (0.7-1.2) | 1.1 (0.2) | 1.1 (0.9-1.2) | 0.019024 |
| Left lateralorbitofrontal | 1.2 (0.4) | 1.1 (0.9-1.4) | 1.4 (0.3) | 1.4 (1.3-1.6) | 0.002713 |
| Left lingual | 0.7 (0.3) | 0.6 (0.5-0.8) | 0.8 (0.3) | 0.7 (0.6-0.8) | 0.011472 |
| Left medialorbitofrontal | 1.1 (0.4) | 1.1 (0.8-1.4) | 1.5 (0.4) | 1.5 (1.2-1.7) | 0.000612 |
| Left middletemporal | 1.1 (0.4) | 1.0 (0.8-1.5) | 1.4 (0.3) | 1.4 (1.2-1.5) | 0.00175 |
| Left parahippocampal | 0.7 (0.2) | 0.7 (0.6-0.9) | 0.9 (0.2) | 0.9 (0.8-1.0) | 0.001626 |
| Left paracentral | 1.0 (0.3) | 0.9 (0.8-1.3) | 1.2 (0.3) | 1.3 (1.1-1.5) | 0.003321 |
| Left parsopercularis | 1.1 (0.4) | 1.1 (0.8-1.4) | 1.4 (0.3) | 1.4 (1.2-1.5) | 0.019983 |
| Left parsorbitalis | 1.2 (0.4) | 1.1 (0.9-1.4) | 1.4 (0.3) | 1.4 (1.3-1.5) | 0.00579 |
| Left parstriangularis | 1.2 (0.4) | 1.2 (1.0-1.5) | 1.5 (0.3) | 1.5 (1.3-1.7) | 0.001492 |
| Left postcentral | 0.9 (0.4) | 0.8 (0.7-1.2) | 1.2 (0.3) | 1.2 (1.0-1.3) | 0.001656 |
| Left posteriorcingulate | 1.2 (0.4) | 1.2 (1.0-1.5) | 1.5 (0.3) | 1.5 (1.3-1.6) | 0.001132 |
| Left precentral | 0.9 (0.3) | 0.8 (0.7-1.2) | 1.1 (0.3) | 1.1 (0.9-1.3) | 0.008997 |
| Left precuneus | 1.3 (0.5) | 1.3 (1.0-1.6) | 1.6 (0.3) | 1.6 (1.4-1.7) | 0.004271 |
| Left rostralanteriorcingulate | 1.2 (0.4) | 1.1 (0.9-1.4) | 1.5 (0.4) | 1.6 (1.4-1.6) | 0.000372 |
| Left rostralmiddlefrontal | 1.3 (0.5) | 1.2 (1.0-1.6) | 1.6 (0.3) | 1.7 (1.4-1.8) | 0.000376 |
| Left superiorfrontal | 1.1 (0.4) | 1.1 (0.8-1.5) | 1.4 (0.3) | 1.5 (1.2-1.6) | 0.001349 |
| Left superiorparietal | 1.1 (0.4) | 1.1 (0.7-1.5) | 1.4 (0.2) | 1.4 (1.2-1.5) | 0.009279 |
| Left superiortemporal | 1.0 (0.3) | 0.9 (0.7-1.2) | 1.2 (0.3) | 1.3 (1.0-1.4) | 0.003681 |
| Left supramarginal | 1.1 (0.4) | 1.1 (0.9-1.4) | 1.4 (0.3) | 1.4 (1.3-1.5) | 0.003827 |
| Left insula | 0.9 (0.3) | 0.9 (0.7-1.1) | 1.1 (0.3) | 1.2 (0.9-1.2) | 0.002247 |
| Right caudalanteriorcingulate | 1.2 (0.4) | 1.1 (1.0-1.4) | 1.4 (0.3) | 1.4 (1.3-1.5) | 0.015409 |
| Right caudalmiddlefrontal | 1.1 (0.4) | 1.2 (0.9-1.4) | 1.4 (0.3) | 1.4 (1.1-1.6) | 0.015376 |
| Right cuneus | 0.8 (0.4) | 0.7 (0.6-0.9) | 0.9 (0.3) | 0.8 (0.7-0.9) | 0.011302 |
| Right fusiform | 1.0 (0.3) | 0.9 (0.7-1.2) | 1.2 (0.3) | 1.2 (1.0-1.3) | 0.004583 |
| Right inferiorparietal | 1.2 (0.4) | 1.2 (0.9-1.5) | 1.4 (0.3) | 1.4 (1.3-1.6) | 0.005234 |
| Right inferiortemporal | 1.1 (0.4) | 1.0 (0.7-1.3) | 1.3 (0.3) | 1.3 (1.1-1.4) | 0.001375 |
| Right isthmuscingulate | 1.1 (0.3) | 1.2 (0.9-1.4) | 1.3 (0.3) | 1.3 (1.2-1.4) | 0.014679 |
| Right lateraloccipital | 1.0 (0.4) | 0.9 (0.7-1.1) | 1.1 (0.3) | 1.1 (0.9-1.3) | 0.011822 |
| Right lateralorbitofrontal | 1.2 (0.4) | 1.2 (0.9-1.5) | 1.4 (0.3) | 1.4 (1.3-1.6) | 0.004475 |
| Right lingual | 0.7 (0.3) | 0.6 (0.5-0.8) | 0.8 (0.3) | 0.8 (0.7-0.8) | 0.011473 |
| Right medialorbitofrontal | 1.1 (0.4) | 1.2 (0.8-1.5) | 1.4 (0.3) | 1.4 (1.3-1.6) | 0.002969 |
| Right middletemporal | 1.1 (0.4) | 1.1 (0.7-1.4) | 1.3 (0.3) | 1.3 (1.2-1.5) | 0.005587 |
| Right parahippocampal | 0.8 (0.2) | 0.8 (0.6-0.9) | 0.9 (0.2) | 1.0 (0.8-1.0) | 0.002309 |
| Right paracentral | 1.0 (0.4) | 0.9 (0.8-1.3) | 1.2 (0.4) | 1.3 (0.9-1.5) | 0.020609 |
| Right parsopercularis | 1.2 (0.4) | 1.2 (0.8-1.4) | 1.4 (0.3) | 1.4 (1.3-1.5) | 0.016969 |
| Right parsorbitalis | 1.2 (0.4) | 1.1 (0.9-1.4) | 1.4 (0.3) | 1.4 (1.2-1.5) | 0.019339 |
| Right parstriangularis | 1.2 (0.4) | 1.2 (0.8-1.6) | 1.5 (0.3) | 1.5 (1.3-1.6) | 0.005772 |
| Right pericalcarine | 0.8 (0.6) | 0.6 (0.4-1.2) | 1.0 (0.6) | 0.9 (0.7-1.2) | 0.044297 |
| Right postcentral | 0.9 (0.4) | 0.8 (0.6-1.2) | 1.1 (0.3) | 1.2 (1.0-1.3) | 0.011474 |
| Right posteriorcingulate | 1.2 (0.4) | 1.2 (0.9-1.6) | 1.5 (0.3) | 1.5 (1.3-1.6) | 0.002382 |
| Right precentral | 0.9 (0.3) | 0.9 (0.7-1.1) | 1.1 (0.3) | 1.1 (0.9-1.2) | 0.02244 |
| Right precuneus | 1.3 (0.5) | 1.4 (0.9-1.6) | 1.5 (0.3) | 1.6 (1.4-1.7) | 0.016464 |
| Right rostralanteriorcingulate | 1.2 (0.4) | 1.2 (0.9-1.4) | 1.4 (0.3) | 1.5 (1.4-1.6) | 0.004459 |
| Right rostralmiddlefrontal | 1.3 (0.4) | 1.3 (0.9-1.6) | 1.6 (0.3) | 1.7 (1.4-1.8) | 0.000853 |
| Right superiorfrontal | 1.1 (0.4) | 1.1 (0.8-1.4) | 1.4 (0.3) | 1.4 (1.2-1.6) | 0.007894 |
| Right superiorparietal | 1.1 (0.4) | 1.0 (0.7-1.4) | 1.3 (0.3) | 1.3 (1.2-1.5) | 0.007204 |
| Right superiortemporal | 0.9 (0.3) | 0.9 (0.6-1.2) | 1.2 (0.3) | 1.2 (1.0-1.3) | 0.005147 |
| Right supramarginal | 1.1 (0.4) | 1.1 (0.8-1.4) | 1.3 (0.3) | 1.3 (1.2-1.4) | 0.012552 |
| Right insula | 0.9 (0.3) | 0.9 (0.7-1.1) | 1.1 (0.3) | 1.2 (0.9-1.3) | 0.001423 |

**B Tau**

| **Region** | **Aff (-)** | | **Aff (+)** | | **p-value** |
| --- | --- | --- | --- | --- | --- |
|  | **Mean (SD)** | **Median (IQR)** | **Mean (SD)** | **Median (IQR)** |  |
| Left caudalmiddlefrontal | 1.4 (0.7) | 1.1 (1.0-1.4) | 1.5 (0.8) | 1.4 (1.1-1.5) | 0.042198 |
| Left entorhinal | 1.7 (0.6) | 1.7 (1.2-2.1) | 2.1 (0.5) | 2.1 (1.9-2.4) | 0.001096 |
| Left fusiform | 1.6 (0.8) | 1.3 (1.2-1.8) | 1.8 (0.6) | 1.5 (1.4-2.2) | 0.004215 |
| Left inferiorparietal | 1.7 (0.8) | 1.3 (1.2-1.7) | 1.9 (0.9) | 1.7 (1.5-2.0) | 0.017001 |
| Left inferiortemporal | 1.8 (0.7) | 1.4 (1.3-2.1) | 2.1 (0.7) | 1.8 (1.7-2.5) | 0.008997 |
| Left isthmuscingulate | 1.4 (0.7) | 1.2 (1.1-1.5) | 1.5 (0.6) | 1.4 (1.2-1.6) | 0.01929 |
| Left lateraloccipital | 1.5 (0.8) | 1.3 (1.2-1.6) | 1.8 (0.9) | 1.5 (1.3-1.8) | 0.020111 |
| Left lingual | 1.2 (0.5) | 1.1 (1.0-1.2) | 1.3 (0.5) | 1.1 (1.1-1.3) | 0.005961 |
| Left middletemporal | 1.8 (0.8) | 1.4 (1.2-1.9) | 2.0 (0.7) | 1.8 (1.5-2.1) | 0.007821 |
| Left parahippocampal | 1.4 (0.5) | 1.3 (1.1-1.6) | 1.6 (0.3) | 1.6 (1.5-1.7) | 0.008858 |
| Left paracentral | 1.0 (0.2) | 1.0 (0.9-1.1) | 1.1 (0.3) | 1.1 (1.0-1.2) | 0.041684 |
| Left pericalcarine | 1.1 (0.2) | 1.1 (1.0-1.2) | 1.1 (0.2) | 1.1 (1.1-1.2) | 0.045913 |
| Left precuneus | 1.5 (0.6) | 1.3 (1.1-1.5) | 1.6 (0.8) | 1.4 (1.3-1.7) | 0.037766 |
| Left superiorfrontal | 1.2 (0.5) | 1.1 (1.0-1.3) | 1.3 (0.5) | 1.2 (1.1-1.4) | 0.038718 |
| Left superiorparietal | 1.4 (0.6) | 1.2 (1.1-1.5) | 1.6 (0.9) | 1.4 (1.2-1.6) | 0.037303 |
| Left superiortemporal | 1.4 (0.5) | 1.2 (1.1-1.5) | 1.5 (0.4) | 1.4 (1.3-1.7) | 0.011999 |
| Right entorhinal | 1.7 (0.6) | 1.6 (1.3-2.2) | 2.1 (0.6) | 2.1 (1.8-2.6) | 0.001205 |
| Right fusiform | 1.7 (0.9) | 1.3 (1.2-2.0) | 1.8 (0.5) | 1.6 (1.4-2.2) | 0.030445 |
| Right inferiortemporal | 1.8 (0.8) | 1.5 (1.3-2.2) | 2.0 (0.6) | 1.8 (1.6-2.7) | 0.021542 |
| Right isthmuscingulate | 1.4 (0.8) | 1.1 (1.0-1.4) | 1.5 (0.7) | 1.4 (1.2-1.6) | 0.005679 |
| Right lateraloccipital | 1.7 (1.1) | 1.2 (1.1-1.6) | 1.7 (0.7) | 1.4 (1.3-2.0) | 0.031237 |
| Right lingual | 1.3 (0.7) | 1.1 (1.0-1.1) | 1.3 (0.4) | 1.2 (1.1-1.4) | 0.007578 |
| Right middletemporal | 1.8 (0.9) | 1.4 (1.3-1.8) | 2.0 (0.7) | 1.7 (1.4-2.4) | 0.032882 |
| Right parahippocampal | 1.4 (0.5) | 1.3 (1.1-1.7) | 1.6 (0.2) | 1.6 (1.4-1.6) | 0.010484 |
| Right precuneus | 1.5 (0.7) | 1.2 (1.1-1.5) | 1.5 (0.8) | 1.3 (1.2-1.5) | 0.042197 |
| Right superiortemporal | 1.4 (0.5) | 1.2 (1.1-1.3) | 1.4 (0.4) | 1.3 (1.2-1.6) | 0.015383 |

**C Aβ and tau**

| **Overlapping regions** | |
| --- | --- |
| Left caudalmiddlefrontal | Left superiorparietal |
| Left fusiform | Left superiortemporal |
| Left inferiorparietal | Right fusiform |
| Left inferiortemporal | Right inferiortemporal |
| Left isthmuscingulate | Right isthmuscingulate |
| Left lateraloccipital | Right lateraloccipital |
| Left lingual | Right lingual |
| Left middletemporal | Right middletemporal |
| Left parahippocampal | Right parahippocampal |
| Left paracentral | Right precuneus |
| Left precuneus | Right superiortemporal |
| Left superiorfrontal |  |

Supplementary Table 3. Regions with significantly different pathology between Aff (+) and Aff (-) (A) Comparison of Aβ, (B) Comparison of tau. (C) Brain regions showing differential Aβ and tau burden, chosen as seed regions for subsequent functional connectivity analysis.

Abbreviations: Aff (-), group without the affective factor; Aff (+), group with the affective factor; SD, standard deviation; IQR, interquartile range

**A Aβ**

| **Region** | **Apa (-)** | | **Apa (+)** | | **p-value** |
| --- | --- | --- | --- | --- | --- |
|  | **Mean (SD)** | **Median (IQR)** | **Mean (SD)** | **Median (IQR)** |  |
| Left caudalanteriorcingulate | 1.2 (0.4) | 1.2 (1.0-1.6) | 1.4 (0.4) | 1.5 (1.2-1.6) | 0.032759 |
| Left caudalmiddlefrontal | 1.2 (0.4) | 1.1 (0.9-1.5) | 1.4 (0.4) | 1.4 (1.3-1.6) | 0.005094 |
| Left cuneus | 0.8 (0.3) | 0.7 (0.6-0.9) | 0.9 (0.3) | 0.8 (0.7-1.1) | 0.027721 |
| Left entorhinal | 0.6 (0.2) | 0.6 (0.5-0.7) | 0.7 (0.2) | 0.7 (0.6-0.8) | 0.014053 |
| Left fusiform | 1.0 (0.3) | 1.0 (0.7-1.2) | 1.2 (0.3) | 1.2 (1.0-1.4) | 0.002624 |
| Left inferiorparietal | 1.2 (0.4) | 1.2 (0.9-1.5) | 1.5 (0.3) | 1.5 (1.2-1.7) | 0.005721 |
| Left inferiortemporal | 1.1 (0.4) | 1.1 (0.8-1.3) | 1.4 (0.4) | 1.4 (1.1-1.6) | 0.005392 |
| Left isthmuscingulate | 1.1 (0.3) | 1.1 (0.9-1.3) | 1.3 (0.3) | 1.3 (1.1-1.5) | 0.008293 |
| Left lateraloccipital | 1.0 (0.4) | 0.9 (0.7-1.1) | 1.1 (0.3) | 1.1 (0.9-1.2) | 0.019632 |
| Left lateralorbitofrontal | 1.2 (0.4) | 1.2 (0.9-1.5) | 1.4 (0.4) | 1.4 (1.3-1.6) | 0.039895 |
| Left lingual | 0.7 (0.3) | 0.6 (0.5-0.7) | 0.8 (0.3) | 0.8 (0.6-1.0) | 0.00207 |
| Left medialorbitofrontal | 1.2 (0.4) | 1.1 (0.9-1.5) | 1.4 (0.4) | 1.4 (1.2-1.7) | 0.029621 |
| Left middletemporal | 1.1 (0.4) | 1.1 (0.8-1.4) | 1.4 (0.3) | 1.5 (1.2-1.6) | 0.004213 |
| Left parahippocampal | 0.7 (0.2) | 0.7 (0.6-0.9) | 0.9 (0.2) | 0.9 (0.8-1.1) | 0.000961 |
| Left paracentral | 1.0 (0.3) | 0.9 (0.7-1.3) | 1.3 (0.3) | 1.4 (1.1-1.5) | 0.000965 |
| Left parsopercularis | 1.1 (0.4) | 1.1 (0.9-1.4) | 1.4 (0.3) | 1.4 (1.2-1.6) | 0.008997 |
| Left parsorbitalis | 1.2 (0.4) | 1.1 (0.9-1.5) | 1.4 (0.3) | 1.4 (1.3-1.5) | 0.011609 |
| Left parstriangularis | 1.3 (0.4) | 1.3 (1.0-1.6) | 1.5 (0.4) | 1.4 (1.3-1.7) | 0.030952 |
| Left pericalcarine | 0.8 (0.5) | 0.7 (0.4-0.9) | 1.0 (0.5) | 1.0 (0.7-1.3) | 0.007443 |
| Left postcentral | 0.9 (0.3) | 0.8 (0.7-1.1) | 1.2 (0.3) | 1.3 (1.0-1.3) | 0.001343 |
| Left posteriorcingulate | 1.2 (0.4) | 1.3 (1.0-1.5) | 1.5 (0.3) | 1.6 (1.3-1.7) | 0.002656 |
| Left precentral | 0.9 (0.3) | 0.8 (0.7-1.1) | 1.1 (0.3) | 1.2 (0.9-1.3) | 0.001818 |
| Left precuneus | 1.3 (0.4) | 1.4 (1.0-1.6) | 1.6 (0.3) | 1.7 (1.5-1.8) | 0.001191 |
| Left rostralanteriorcingulate | 1.2 (0.4) | 1.2 (0.9-1.5) | 1.4 (0.4) | 1.4 (1.2-1.7) | 0.018866 |
| Left rostralmiddlefrontal | 1.3 (0.4) | 1.3 (1.0-1.7) | 1.6 (0.4) | 1.5 (1.4-1.8) | 0.011585 |
| Left superiorfrontal | 1.1 (0.4) | 1.1 (0.8-1.5) | 1.4 (0.4) | 1.5 (1.2-1.6) | 0.010343 |
| Left superiorparietal | 1.1 (0.4) | 1.1 (0.8-1.4) | 1.4 (0.3) | 1.4 (1.2-1.6) | 0.000726 |
| Left superiortemporal | 1.0 (0.3) | 0.9 (0.7-1.2) | 1.2 (0.3) | 1.3 (1.0-1.5) | 0.002647 |
| Left supramarginal | 1.1 (0.4) | 1.1 (0.9-1.4) | 1.4 (0.3) | 1.4 (1.3-1.6) | 0.001113 |
| Left transversetemporal | 0.9 (0.5) | 0.8 (0.6-1.3) | 1.2 (0.4) | 1.3 (0.9-1.5) | 0.006795 |
| Left insula | 0.9 (0.3) | 0.9 (0.7-1.1) | 1.1 (0.3) | 1.2 (1.0-1.3) | 0.002096 |
| Right caudalanteriorcingulate | 1.2 (0.4) | 1.2 (0.9-1.4) | 1.4 (0.3) | 1.3 (1.2-1.6) | 0.018124 |
| Right caudalmiddlefrontal | 1.2 (0.4) | 1.2 (0.9-1.4) | 1.4 (0.4) | 1.3 (1.1-1.6) | 0.019177 |
| Right cuneus | 0.8 (0.3) | 0.7 (0.6-0.9) | 0.9 (0.3) | 0.8 (0.7-1.0) | 0.040909 |
| Right fusiform | 1.0 (0.3) | 1.0 (0.7-1.2) | 1.1 (0.3) | 1.2 (0.9-1.3) | 0.024873 |
| Right inferiorparietal | 1.2 (0.4) | 1.3 (0.9-1.5) | 1.4 (0.4) | 1.4 (1.1-1.6) | 0.032311 |
| Right inferiortemporal | 1.1 (0.4) | 1.1 (0.8-1.3) | 1.3 (0.3) | 1.3 (1.1-1.5) | 0.007207 |
| Right isthmuscingulate | 1.1 (0.3) | 1.2 (0.9-1.3) | 1.4 (0.3) | 1.3 (1.2-1.5) | 0.004663 |
| Right lateralorbitofrontal | 1.2 (0.3) | 1.2 (0.9-1.5) | 1.4 (0.4) | 1.4 (1.2-1.7) | 0.026331 |
| Right lingual | 0.7 (0.3) | 0.6 (0.5-0.8) | 0.8 (0.3) | 0.8 (0.6-1.0) | 0.021664 |
| Right medialorbitofrontal | 1.2 (0.4) | 1.2 (0.9-1.5) | 1.4 (0.4) | 1.4 (1.3-1.7) | 0.00622 |
| Right middletemporal | 1.1 (0.4) | 1.1 (0.8-1.3) | 1.4 (0.4) | 1.4 (1.2-1.6) | 0.00437 |
| Right parahippocampal | 0.8 (0.2) | 0.8 (0.6-0.9) | 0.9 (0.2) | 1.0 (0.8-1.1) | 0.003237 |
| Right paracentral | 1.0 (0.3) | 0.9 (0.8-1.3) | 1.3 (0.4) | 1.4 (1.0-1.5) | 0.003742 |
| Right parsopercularis | 1.2 (0.4) | 1.2 (0.8-1.5) | 1.4 (0.3) | 1.3 (1.2-1.5) | 0.024874 |
| Right parstriangularis | 1.2 (0.4) | 1.3 (0.9-1.6) | 1.5 (0.4) | 1.5 (1.3-1.7) | 0.014914 |
| Right pericalcarine | 0.8 (0.6) | 0.6 (0.4-1.2) | 1.1 (0.6) | 1.0 (0.6-1.5) | 0.031657 |
| Right postcentral | 0.9 (0.3) | 0.8 (0.6-1.2) | 1.2 (0.3) | 1.2 (1.0-1.3) | 0.003613 |
| Right posteriorcingulate | 1.2 (0.4) | 1.3 (0.9-1.5) | 1.5 (0.4) | 1.5 (1.3-1.7) | 0.003541 |
| Right precentral | 0.9 (0.3) | 0.9 (0.7-1.1) | 1.1 (0.3) | 1.1 (0.9-1.3) | 0.011205 |
| Right precuneus | 1.3 (0.4) | 1.4 (0.9-1.6) | 1.6 (0.4) | 1.5 (1.4-1.8) | 0.005146 |
| Right rostralanteriorcingulate | 1.2 (0.4) | 1.2 (0.9-1.5) | 1.4 (0.4) | 1.4 (1.3-1.7) | 0.042222 |
| Right rostralmiddlefrontal | 1.3 (0.4) | 1.4 (0.9-1.7) | 1.6 (0.4) | 1.6 (1.4-1.8) | 0.018724 |
| Right superiorfrontal | 1.2 (0.4) | 1.1 (0.8-1.4) | 1.4 (0.4) | 1.4 (1.2-1.6) | 0.008514 |
| Right superiorparietal | 1.1 (0.4) | 1.1 (0.8-1.4) | 1.3 (0.4) | 1.3 (1.1-1.6) | 0.029117 |
| Right superiortemporal | 0.9 (0.3) | 0.9 (0.6-1.2) | 1.2 (0.3) | 1.2 (1.0-1.4) | 0.002486 |
| Right supramarginal | 1.1 (0.3) | 1.1 (0.8-1.4) | 1.4 (0.3) | 1.3 (1.2-1.5) | 0.007564 |
| Right transversetemporal | 0.9 (0.5) | 0.8 (0.6-1.2) | 1.2 (0.4) | 1.2 (0.9-1.3) | 0.011007 |
| Right insula | 0.9 (0.3) | 0.9 (0.7-1.1) | 1.1 (0.3) | 1.1 (0.9-1.3) | 0.00501 |

**B Tau**

| **Region** | **Apa (-)** | | **Apa (+)** | | **p-value** |
| --- | --- | --- | --- | --- | --- |
|  | **Mean (SD)** | **Median (IQR)** | **Mean (SD)** | **Median (IQR)** |  |
| Left entorhinal | 1.8 (0.6) | 1.7 (1.2-2.1) | 2.0 (0.5) | 2.0 (1.6-2.3) | 0.032492 |
| Left fusiform | 1.6 (0.8) | 1.3 (1.2-1.8) | 1.8 (0.6) | 1.8 (1.4-2.1) | 0.017259 |
| Left inferiorparietal | 1.7 (0.8) | 1.3 (1.2-1.7) | 1.9 (0.9) | 1.8 (1.5-2.0) | 0.010536 |
| Left inferiortemporal | 1.8 (0.8) | 1.5 (1.3-2.0) | 2.1 (0.6) | 1.9 (1.7-2.4) | 0.009014 |
| Left isthmuscingulate | 1.4 (0.6) | 1.2 (1.1-1.5) | 1.6 (0.6) | 1.4 (1.1-1.7) | 0.014266 |
| Left middletemporal | 1.8 (0.8) | 1.4 (1.3-1.8) | 2.0 (0.7) | 1.9 (1.6-2.2) | 0.003741 |
| Left posteriorcingulate | 1.3 (0.5) | 1.2 (1.1-1.4) | 1.5 (0.5) | 1.4 (1.2-1.8) | 0.032073 |
| Left precuneus | 1.4 (0.7) | 1.3 (1.2-1.4) | 1.6 (0.6) | 1.4 (1.2-1.7) | 0.045175 |
| Left superiortemporal | 1.4 (0.5) | 1.2 (1.1-1.4) | 1.5 (0.3) | 1.5 (1.3-1.6) | 0.018022 |
| Left supramarginal | 1.4 (0.6) | 1.2 (1.1-1.4) | 1.6 (0.6) | 1.4 (1.2-1.8) | 0.018546 |
| Right entorhinal | 1.8 (0.6) | 1.6 (1.3-2.2) | 2.0 (0.5) | 2.1 (1.6-2.5) | 0.027351 |
| Right fusiform | 1.7 (0.8) | 1.4 (1.2-1.8) | 1.9 (0.7) | 1.9 (1.4-2.2) | 0.02197 |
| Right inferiorparietal | 1.7 (0.9) | 1.3 (1.2-1.9) | 2.0 (1.1) | 1.6 (1.4-2.2) | 0.01285 |
| Right inferiortemporal | 1.8 (0.8) | 1.5 (1.3-2.2) | 2.1 (0.7) | 1.8 (1.6-2.6) | 0.007811 |
| Right isthmuscingulate | 1.4 (0.7) | 1.1 (1.1-1.4) | 1.7 (0.8) | 1.5 (1.2-1.6) | 0.006324 |
| Right middletemporal | 1.8 (0.8) | 1.4 (1.3-1.8) | 2.1 (0.9) | 1.7 (1.5-2.5) | 0.003613 |
| Right parahippocampal | 1.4 (0.4) | 1.3 (1.1-1.6) | 1.7 (0.4) | 1.6 (1.5-1.9) | 0.00282 |
| Right posteriorcingulate | 1.4 (0.5) | 1.2 (1.1-1.4) | 1.6 (0.8) | 1.4 (1.2-1.7) | 0.024202 |
| Right precuneus | 1.4 (0.7) | 1.2 (1.1-1.4) | 1.7 (0.8) | 1.4 (1.3-1.6) | 0.021067 |
| Right superiortemporal | 1.4 (0.5) | 1.2 (1.1-1.4) | 1.4 (0.4) | 1.3 (1.2-1.6) | 0.021967 |
| Right supramarginal | 1.4 (0.5) | 1.2 (1.1-1.5) | 1.6 (0.7) | 1.4 (1.2-1.7) | 0.018021 |

**C Aβ and tau**

| **Overlapping regions** | |
| --- | --- |
| Left entorhinal | Right fusiform |
| Left fusiform | Right inferiorparietal |
| Left inferiorparietal | Right inferiortemporal |
| Left inferiortemporal | Right isthmuscingulate |
| Left isthmuscingulate | Right middletemporal |
| Left middletemporal | Right parahippocampal |
| Left posteriorcingulate | Right posteriorcingulate |
| Left precuneus | Right precuneus |
| Left superiortemporal | Right superiortemporal |
| Left supramarginal | Right supramarginal |

Supplementary Table 4. Regions with significantly different pathology between Apa (+) and Apa (-) (A) Comparison of Aβ, (B) Comparison of tau. (C) Brain regions showing differential Aβ and tau burden, chosen as seed regions for subsequent functional connectivity analysis.

Abbreviations: Apa (-), group without the apathy factor; Apa (+), group with the apathy factor; SD, standard deviation; IQR, interquartile range

**A Aβ**

| **Region** | **Hyp (-)** | | **Hyp (+)** | | **p-value** |
| --- | --- | --- | --- | --- | --- |
|  | **Mean (SD)** | **Median (IQR)** | **Mean (SD)** | **Median (IQR)** |  |
| Left caudalanteriorcingulate | 1.2 (0.4) | 1.2 (1.0-1.5) | 1.5 (0.3) | 1.5 (1.3-1.6) | 0.008622 |
| Left caudalmiddlefrontal | 1.2 (0.4) | 1.1 (0.9-1.5) | 1.4 (0.4) | 1.4 (1.1-1.6) | 0.015059 |
| Left cuneus | 0.8 (0.3) | 0.7 (0.6-0.9) | 0.9 (0.3) | 0.9 (0.7-1.2) | 0.009538 |
| Left entorhinal | 0.6 (0.2) | 0.6 (0.5-0.7) | 0.7 (0.2) | 0.7 (0.6-0.8) | 0.010947 |
| Left fusiform | 1.0 (0.3) | 0.9 (0.7-1.2) | 1.2 (0.3) | 1.1 (1.0-1.4) | 0.005537 |
| Left inferiorparietal | 1.2 (0.4) | 1.2 (0.9-1.5) | 1.5 (0.3) | 1.4 (1.2-1.7) | 0.003758 |
| Left inferiortemporal | 1.1 (0.4) | 1.1 (0.8-1.4) | 1.3 (0.3) | 1.3 (1.1-1.6) | 0.008391 |
| Left isthmuscingulate | 1.1 (0.3) | 1.1 (0.8-1.3) | 1.3 (0.2) | 1.3 (1.1-1.4) | 0.008436 |
| Left lateraloccipital | 1.0 (0.4) | 0.9 (0.7-1.1) | 1.2 (0.3) | 1.1 (0.9-1.3) | 0.010949 |
| Left lateralorbitofrontal | 1.2 (0.4) | 1.1 (0.9-1.4) | 1.4 (0.3) | 1.4 (1.3-1.6) | 0.005918 |
| Left lingual | 0.7 (0.3) | 0.6 (0.5-0.8) | 0.8 (0.3) | 0.8 (0.6-0.9) | 0.007778 |
| Left medialorbitofrontal | 1.1 (0.4) | 1.1 (0.8-1.5) | 1.4 (0.3) | 1.4 (1.1-1.7) | 0.003158 |
| Left middletemporal | 1.1 (0.4) | 1.1 (0.8-1.5) | 1.4 (0.3) | 1.4 (1.1-1.6) | 0.00728 |
| Left parahippocampal | 0.8 (0.2) | 0.7 (0.6-0.9) | 0.9 (0.2) | 0.9 (0.7-1.0) | 0.031677 |
| Left paracentral | 1.0 (0.3) | 0.9 (0.7-1.3) | 1.3 (0.3) | 1.3 (1.0-1.5) | 0.001473 |
| Left parsopercularis | 1.1 (0.4) | 1.1 (0.9-1.4) | 1.4 (0.3) | 1.4 (1.2-1.6) | 0.005254 |
| Left parsorbitalis | 1.2 (0.4) | 1.1 (0.9-1.5) | 1.4 (0.3) | 1.4 (1.3-1.5) | 0.008255 |
| Left parstriangularis | 1.3 (0.4) | 1.2 (1.0-1.6) | 1.5 (0.3) | 1.5 (1.3-1.7) | 0.018837 |
| Left pericalcarine | 0.8 (0.5) | 0.7 (0.4-0.9) | 1.0 (0.5) | 1.0 (0.6-1.4) | 0.012541 |
| Left postcentral | 0.9 (0.3) | 0.9 (0.7-1.1) | 1.2 (0.3) | 1.3 (1.0-1.3) | 0.000929 |
| Left posteriorcingulate | 1.2 (0.4) | 1.2 (0.9-1.5) | 1.5 (0.3) | 1.6 (1.3-1.7) | 0.001593 |
| Left precentral | 0.9 (0.3) | 0.8 (0.7-1.1) | 1.1 (0.3) | 1.2 (0.9-1.3) | 0.003158 |
| Left precuneus | 1.3 (0.4) | 1.4 (1.0-1.6) | 1.6 (0.3) | 1.6 (1.4-1.8) | 0.002473 |
| Left rostralanteriorcingulate | 1.2 (0.4) | 1.1 (0.9-1.5) | 1.5 (0.4) | 1.4 (1.3-1.7) | 0.002132 |
| Left rostralmiddlefrontal | 1.3 (0.4) | 1.3 (1.0-1.7) | 1.6 (0.3) | 1.6 (1.4-1.8) | 0.00577 |
| Left superiorfrontal | 1.1 (0.4) | 1.1 (0.8-1.4) | 1.4 (0.3) | 1.5 (1.2-1.7) | 0.002232 |
| Left superiorparietal | 1.1 (0.4) | 1.2 (0.8-1.4) | 1.4 (0.3) | 1.4 (1.2-1.6) | 0.008147 |
| Left superiortemporal | 1.0 (0.3) | 0.9 (0.7-1.3) | 1.2 (0.3) | 1.1 (1.0-1.4) | 0.004766 |
| Left supramarginal | 1.1 (0.4) | 1.1 (0.8-1.4) | 1.4 (0.3) | 1.4 (1.3-1.6) | 0.002756 |
| Left transversetemporal | 0.9 (0.5) | 0.8 (0.6-1.3) | 1.2 (0.4) | 1.3 (1.0-1.5) | 0.008385 |
| Left insula | 0.9 (0.3) | 0.8 (0.7-1.1) | 1.1 (0.2) | 1.1 (0.9-1.2) | 0.002695 |
| Right caudalanteriorcingulate | 1.2 (0.3) | 1.1 (0.9-1.4) | 1.4 (0.3) | 1.4 (1.3-1.6) | 0.001601 |
| Right caudalmiddlefrontal | 1.1 (0.4) | 1.1 (0.9-1.4) | 1.4 (0.3) | 1.4 (1.2-1.6) | 0.00388 |
| Right cuneus | 0.8 (0.3) | 0.7 (0.6-0.9) | 1.0 (0.4) | 0.8 (0.7-1.2) | 0.015412 |
| Right fusiform | 1.0 (0.3) | 0.9 (0.7-1.1) | 1.2 (0.2) | 1.2 (1.1-1.4) | 0.000423 |
| Right inferiorparietal | 1.2 (0.4) | 1.2 (0.8-1.4) | 1.5 (0.3) | 1.5 (1.3-1.7) | 0.00093 |
| Right inferiortemporal | 1.1 (0.4) | 1.0 (0.7-1.3) | 1.4 (0.3) | 1.4 (1.1-1.5) | 0.000219 |
| Right isthmuscingulate | 1.1 (0.3) | 1.2 (0.9-1.4) | 1.4 (0.3) | 1.3 (1.2-1.5) | 0.001872 |
| Right lateraloccipital | 1.0 (0.4) | 0.9 (0.7-1.1) | 1.2 (0.3) | 1.1 (1.0-1.4) | 0.001473 |
| Right lateralorbitofrontal | 1.2 (0.3) | 1.2 (0.9-1.5) | 1.5 (0.3) | 1.4 (1.3-1.6) | 0.002803 |
| Right lingual | 0.7 (0.3) | 0.6 (0.5-0.8) | 0.9 (0.3) | 0.8 (0.6-1.1) | 0.004163 |
| Right medialorbitofrontal | 1.1 (0.4) | 1.2 (0.8-1.5) | 1.5 (0.3) | 1.4 (1.3-1.7) | 0.000232 |
| Right middletemporal | 1.1 (0.4) | 1.1 (0.7-1.3) | 1.4 (0.3) | 1.4 (1.2-1.6) | 0.000151 |
| Right parahippocampal | 0.8 (0.2) | 0.8 (0.6-0.9) | 0.9 (0.2) | 1.0 (0.8-1.1) | 0.00057 |
| Right paracentral | 1.0 (0.3) | 0.9 (0.8-1.2) | 1.3 (0.3) | 1.4 (1.1-1.5) | 0.001366 |
| Right parsopercularis | 1.1 (0.4) | 1.2 (0.8-1.5) | 1.4 (0.3) | 1.4 (1.3-1.6) | 0.00327 |
| Right parsorbitalis | 1.2 (0.3) | 1.2 (0.9-1.4) | 1.4 (0.3) | 1.3 (1.2-1.5) | 0.016137 |
| Right parstriangularis | 1.2 (0.4) | 1.2 (0.8-1.6) | 1.5 (0.3) | 1.5 (1.3-1.6) | 0.002791 |
| Right pericalcarine | 0.8 (0.5) | 0.7 (0.4-1.1) | 1.1 (0.6) | 1.0 (0.6-1.6) | 0.020773 |
| Right postcentral | 0.9 (0.3) | 0.8 (0.6-1.1) | 1.2 (0.3) | 1.2 (1.1-1.3) | 9.59E-05 |
| Right posteriorcingulate | 1.2 (0.4) | 1.2 (0.9-1.5) | 1.5 (0.4) | 1.5 (1.3-1.7) | 0.003353 |
| Right precentral | 0.9 (0.3) | 0.8 (0.7-1.1) | 1.1 (0.3) | 1.1 (1.0-1.3) | 0.000489 |
| Right precuneus | 1.3 (0.4) | 1.4 (0.9-1.6) | 1.6 (0.3) | 1.6 (1.3-1.8) | 0.000593 |
| Right rostralanteriorcingulate | 1.2 (0.4) | 1.2 (0.9-1.5) | 1.5 (0.3) | 1.5 (1.4-1.7) | 8.42E-05 |
| Right rostralmiddlefrontal | 1.3 (0.4) | 1.3 (0.9-1.6) | 1.7 (0.3) | 1.7 (1.5-1.8) | 0.001175 |
| Right superiorfrontal | 1.1 (0.4) | 1.1 (0.8-1.4) | 1.5 (0.3) | 1.5 (1.3-1.6) | 0.00054 |
| Right superiorparietal | 1.1 (0.4) | 1.0 (0.7-1.4) | 1.4 (0.3) | 1.4 (1.2-1.6) | 0.000461 |
| Right superiortemporal | 0.9 (0.3) | 0.9 (0.6-1.2) | 1.2 (0.3) | 1.2 (1.0-1.4) | 0.000157 |
| Right supramarginal | 1.1 (0.3) | 1.1 (0.8-1.3) | 1.4 (0.3) | 1.4 (1.3-1.5) | 0.000126 |
| Right transversetemporal | 0.9 (0.5) | 0.8 (0.5-1.2) | 1.2 (0.3) | 1.2 (1.0-1.3) | 0.000318 |
| Right insula | 0.9 (0.3) | 0.9 (0.7-1.1) | 1.1 (0.2) | 1.1 (1.1-1.2) | 0.000184 |

**B Tau**

| **Region** | **Hyp (-)** | | **Hyp (+)** | | **p-value** |
| --- | --- | --- | --- | --- | --- |
|  | **Mean (SD)** | **Median (IQR)** | **Mean (SD)** | **Median (IQR)** |  |
| Left caudalanteriorcingulate | 1.2 (0.4) | 1.2 (1.0-1.3) | 1.1 (0.2) | 1.1 (1.0-1.2) | 0.031097 |
| Right caudalanteriorcingulate | 1.2 (0.3) | 1.1 (1.0-1.3) | 1.1 (0.2) | 1.0 (0.9-1.2) | 0.018048 |
| Right inferiorparietal | 1.7 (0.9) | 1.3 (1.2-1.9) | 2.0 (1.1) | 1.6 (1.3-2.1) | 0.026549 |
| Right lateraloccipital | 1.7 (1.0) | 1.3 (1.1-1.5) | 1.8 (0.8) | 1.5 (1.2-2.1) | 0.036278 |
| Right middletemporal | 1.8 (0.9) | 1.4 (1.3-1.9) | 2.0 (0.8) | 1.7 (1.4-2.4) | 0.014754 |

**C Aβ and tau**

| **Overlapping regions** | |
| --- | --- |
| Right inferiorparietal | Left caudalanteriorcingulate |
| Right lateraloccipital | Right caudalanteriorcingulate |
| Right middletemporal |  |

Supplementary Table 5. Regions with significantly different pathology between Hyp (+) and Hyp (-) (A) Comparison of Aβ, (B) Comparison of tau. In caudal anterior cingulate of both hemispheres, Hyp (+) had lower mean or median value in tau accumulation. (C) Brain regions showing differential Aβ and tau burden, chosen as seed regions for subsequent functional connectivity analysis (except for bilateral caudal anterior cingulate).

Abbreviations: Hyp (-), group without the hyperactivity factor; Hyp (+), group with the hyperactivity factor; SD, standard deviation; IQR, interquartile range

| **Variables** | **β** | **Standard error** | **p-Value** |
| --- | --- | --- | --- |
| Intercept | 25.44678 | 13.4108 | 0.0786 |
| Age | -0.09876 | 0.1232 | 0.4362 |
| Sex | 2.95289 | 1.63853 | 0.0931 |
| Education | 0.20972 | 0.32303 | 0.5267 |
| MMSE | -0.34003 | 0.23631 | 0.1721 |
| Connectivity with rFFG | -4.85227 | 8.54975 | 0.5793 |
| Tau SUVR in rFFG | -4.77326 | 4.35426 | 0.2915 |
| Amyloid SUVR in rFFG | -10.3982 | 8.62423 | 0.2479 |
| Tau : Amyloid SUVR | 4.94409 | 3.62177 | 0.1938 |

**Supplementary Table 6. Generalized linear model to predict the severity of the apathy factor with rFFG**

**Abbreviations:** MMSE, Mini-Mental State Examination; rFFG, right fusiform gyrus; SUVR, standardized uptake value ratio; Tau : Amyloid SUVR, the interaction term of tau and amyloid SUVR in the rFFG

| **Variables** | **β** | **Standard error** | **p-Value** |
| --- | --- | --- | --- |
| Intercept | 24.7271 | 20.141 | 0.2398 |
| Age | -0.1521 | 0.1242 | 0.2408 |
| Sex | 2.3646 | 1.6655 | 0.1776 |
| Education | 0.3712 | 0.4166 | 0.388 |
| MMSE | -0.5519 | 0.2914 | 0.0791 |
| Connectivity with rPH | -11.4316 | 11.1472 | 0.3225 |
| Tau SUVR in rPH | -1.948 | 7.4865 | 0.7985 |
| Amyloid SUVR in rPH | -5.1915 | 18.1865 | 0.7795 |
| Tau : Amyloid SUVR | 3.3673 | 9.3373 | 0.7238 |

**Supplementary Table 7. Generalized linear model to predict the severity of the apathy factor with rPH**

**Abbreviations:** MMSE, Mini-Mental State Examination; rPH, right parahippocampal gyrus; SUVR, standardized uptake value ratio; Tau : Amyloid SUVR, the interaction term of tau and amyloid SUVR in the rPH

| **Variables** | **β** | **Standard error** | **p-Value** |
| --- | --- | --- | --- |
| Intercept | 4.71738 | 20.99524 | 0.8267 |
| Age | -0.09097 | 0.12954 | 0.4985 |
| Sex | 3.27837 | 1.71312 | 0.0847 |
| Education | 0.40733 | 0.39338 | 0.3248 |
| MMSE | -0.28467 | 0.31986 | 0.3944 |
| Connectivity with rFFG | -8.89161 | 8.80673 | 0.3365 |
| Tau SUVR in rFFG | -3.94339 | 5.87466 | 0.5173 |
| Amyloid SUVR in rFFG | 7.97766 | 13.48341 | 0.5672 |
| Tau : Amyloid SUVR in rFFG | 3.51993 | 4.65424 | 0.4669 |
| Connectivity with rPH | -7.3784 | 10.77205 | 0.5089 |
| Tau SUVR in rPH | 6.90589 | 9.03462 | 0.4623 |
| Amyloid SUVR in rPH | -0.17159 | 19.96684 | 0.9933 |
| Tau : Amyloid SUVR in rPH | -9.70009 | 11.01728 | 0.3993 |

**Supplementary Table 8. Generalized linear model to predict the severity of the apathy factor with both rFFG and rPH**

**Abbreviations:** MMSE, Mini-Mental State Examination; rFFG, right fusiform gyrus; rPH, right parahippocampal gyrus; SUVR, standardized uptake value ratio; Tau : Amyloid SUVR, the interaction term of tau and amyloid SUVR

**A Aβ (MCI)**

| **Region** | **NPS (-)** | | **NPS (+)** | | **p-value** |
| --- | --- | --- | --- | --- | --- |
|  | **Mean (SD)** | **Median (IQR)** | **Mean (SD)** | **Median (IQR)** |  |
| Left caudalmiddlefrontal | 1.0 (0.4) | 1.0 (0.7-1.2) | 1.3 (0.3) | 1.3 (1.0-1.5) | 0.024304 |
| Left fusiform | 0.9 (0.4) | 0.8 (0.7-1.1) | 1.1 (0.3) | 1.1 (1.0-1.3) | 0.004249 |
| Left inferiorparietal | 1.0 (0.4) | 0.9 (0.8-1.2) | 1.3 (0.3) | 1.3 (1.2-1.5) | 0.002779 |
| Left inferiortemporal | 1.0 (0.4) | 0.8 (0.7-1.2) | 1.2 (0.3) | 1.2 (1.1-1.4) | 0.003443 |
| Left isthmuscingulate | 1.0 (0.4) | 1.0 (0.8-1.3) | 1.2 (0.3) | 1.3 (1.1-1.4) | 0.040145 |
| Left lateraloccipital | 0.9 (0.4) | 0.7 (0.6-1.2) | 1.0 (0.3) | 0.9 (0.9-1.2) | 0.037069 |
| Left lateralorbitofrontal | 1.1 (0.4) | 1.0 (0.8-1.4) | 1.3 (0.3) | 1.3 (1.1-1.5) | 0.02196 |
| Left lingual | 0.6 (0.4) | 0.5 (0.5-0.7) | 0.8 (0.3) | 0.8 (0.6-0.8) | 0.012415 |
| Left medialorbitofrontal | 1.0 (0.4) | 1.1 (0.8-1.3) | 1.3 (0.4) | 1.4 (1.1-1.6) | 0.006359 |
| Left middletemporal | 1.0 (0.4) | 0.9 (0.7-1.2) | 1.3 (0.3) | 1.2 (1.1-1.5) | 0.003963 |
| Left parahippocampal | 0.7 (0.2) | 0.6 (0.5-0.7) | 0.9 (0.2) | 0.9 (0.7-1.0) | 0.001786 |
| Left paracentral | 0.9 (0.3) | 0.8 (0.7-1.0) | 1.1 (0.3) | 1.1 (0.9-1.4) | 0.021654 |
| Left parsopercularis | 1.1 (0.4) | 1.1 (0.8-1.3) | 1.3 (0.3) | 1.4 (1.0-1.5) | 0.042573 |
| Left postcentral | 0.8 (0.3) | 0.7 (0.6-1.0) | 1.0 (0.3) | 1.1 (0.7-1.3) | 0.008818 |
| Left posteriorcingulate | 1.1 (0.4) | 1.1 (0.9-1.3) | 1.4 (0.3) | 1.5 (1.2-1.6) | 0.021513 |
| Left precentral | 0.8 (0.2) | 0.8 (0.7-0.9) | 1.0 (0.3) | 1.0 (0.8-1.2) | 0.027006 |
| Left precuneus | 1.2 (0.4) | 1.0 (0.8-1.4) | 1.5 (0.3) | 1.5 (1.3-1.7) | 0.008144 |
| Left rostralanteriorcingulate | 1.1 (0.4) | 1.0 (0.8-1.3) | 1.4 (0.3) | 1.4 (1.1-1.6) | 0.013206 |
| Left rostralmiddlefrontal | 1.2 (0.4) | 1.2 (0.9-1.5) | 1.5 (0.3) | 1.5 (1.4-1.8) | 0.00416 |
| Left superiorfrontal | 1.0 (0.4) | 0.9 (0.8-1.3) | 1.3 (0.3) | 1.3 (1.1-1.6) | 0.004343 |
| Left superiorparietal | 1.0 (0.4) | 0.9 (0.7-1.1) | 1.3 (0.3) | 1.3 (1.1-1.5) | 0.002317 |
| Left superiortemporal | 0.9 (0.3) | 0.8 (0.6-1.0) | 1.1 (0.3) | 1.1 (0.9-1.3) | 0.016957 |
| Left supramarginal | 1.0 (0.4) | 0.9 (0.8-1.1) | 1.3 (0.3) | 1.3 (1.0-1.5) | 0.002457 |
| Left transversetemporal | 0.9 (0.5) | 0.7 (0.6-0.9) | 1.1 (0.4) | 1.1 (0.8-1.4) | 0.01722 |
| Left insula | 0.8 (0.3) | 0.8 (0.6-0.9) | 1.0 (0.3) | 1.0 (0.9-1.2) | 0.009802 |
| Right caudalmiddlefrontal | 1.0 (0.3) | 0.9 (0.8-1.2) | 1.3 (0.3) | 1.3 (1.1-1.5) | 0.028481 |
| Right fusiform | 0.9 (0.4) | 0.8 (0.6-1.0) | 1.1 (0.2) | 1.1 (0.9-1.2) | 0.005768 |
| Right inferiorparietal | 1.1 (0.4) | 1.0 (0.8-1.3) | 1.3 (0.3) | 1.3 (1.2-1.5) | 0.049096 |
| Right inferiortemporal | 0.9 (0.3) | 0.8 (0.7-1.1) | 1.2 (0.3) | 1.2 (1.0-1.4) | 0.001317 |
| Right lateraloccipital | 0.9 (0.5) | 0.7 (0.7-1.0) | 1.0 (0.3) | 1.0 (0.9-1.1) | 0.021654 |
| Right lateralorbitofrontal | 1.1 (0.3) | 1.1 (0.8-1.4) | 1.3 (0.3) | 1.3 (1.1-1.6) | 0.044613 |
| Right lingual | 0.7 (0.4) | 0.6 (0.5-0.6) | 0.8 (0.3) | 0.7 (0.6-0.8) | 0.022892 |
| Right medialorbitofrontal | 1.0 (0.4) | 1.1 (0.8-1.3) | 1.3 (0.3) | 1.3 (1.2-1.5) | 0.012154 |
| Right middletemporal | 1.0 (0.4) | 0.9 (0.7-1.1) | 1.2 (0.3) | 1.2 (1.1-1.4) | 0.004395 |
| Right parahippocampal | 0.7 (0.2) | 0.7 (0.5-0.8) | 0.9 (0.2) | 0.9 (0.7-1.0) | 0.002284 |
| Right postcentral | 0.8 (0.4) | 0.7 (0.6-1.0) | 1.0 (0.3) | 1.1 (0.8-1.2) | 0.022266 |
| Right rostralanteriorcingulate | 1.1 (0.3) | 1.0 (0.9-1.3) | 1.3 (0.4) | 1.4 (1.2-1.5) | 0.018981 |
| Right rostralmiddlefrontal | 1.2 (0.4) | 1.2 (0.8-1.5) | 1.5 (0.3) | 1.5 (1.3-1.8) | 0.011168 |
| Right superiorfrontal | 1.0 (0.3) | 1.0 (0.8-1.3) | 1.3 (0.3) | 1.3 (1.1-1.5) | 0.01578 |
| Right superiorparietal | 1.0 (0.4) | 0.9 (0.7-1.0) | 1.2 (0.3) | 1.2 (1.0-1.4) | 0.006809 |
| Right superiortemporal | 0.8 (0.3) | 0.7 (0.6-1.0) | 1.0 (0.3) | 1.0 (0.9-1.3) | 0.008537 |
| Right supramarginal | 1.0 (0.4) | 0.9 (0.7-1.3) | 1.2 (0.3) | 1.3 (1.1-1.4) | 0.021317 |
| Right transversetemporal | 0.8 (0.5) | 0.7 (0.5-0.9) | 1.0 (0.4) | 1.0 (0.7-1.3) | 0.043142 |
| Right insula | 0.8 (0.2) | 0.7 (0.7-1.0) | 1.0 (0.3) | 1.1 (0.9-1.2) | 0.007881 |

Supplementary Table 9. Regions with significantly different pathological accumulation between NPS (+) and NPS (-) (A) Aβ accumulation differences in MCI participants. No significant differences in tau accumulation were observed in MCI participants, nor in Aβ or tau accumulation in AD participants.

Abbreviations: NPS (-), group without any NPS; NPS (+), group with at least one NPS; MCI, mild cognitive impairment; SD, standard deviation; IQR, interquartile range

**A Aβ (MCI)**

| **Region** | **Aff (-)** | | **Aff (+)** | | **p-value** |
| --- | --- | --- | --- | --- | --- |
|  | **Mean (SD)** | **Median (IQR)** | **Mean (SD)** | **Median (IQR)** |  |
| Left caudalmiddlefrontal | 1.1 (0.4) | 1.0 (0.8-1.4) | 1.3 (0.3) | 1.4 (1.2-1.5) | 0.0419 |
| Left fusiform | 1.0 (0.3) | 0.8 (0.7-1.1) | 1.2 (0.3) | 1.2 (1.0-1.3) | 0.01485 |
| Left inferiortemporal | 1.0 (0.3) | 0.9 (0.8-1.2) | 1.3 (0.4) | 1.2 (1.1-1.5) | 0.014108 |
| Left isthmuscingulate | 1.1 (0.3) | 1.1 (0.9-1.3) | 1.3 (0.3) | 1.3 (1.2-1.5) | 0.034666 |
| Left lingual | 0.7 (0.4) | 0.6 (0.4-0.7) | 0.8 (0.3) | 0.8 (0.6-0.8) | 0.00854 |
| Left medialorbitofrontal | 1.1 (0.4) | 1.1 (0.9-1.3) | 1.4 (0.4) | 1.4 (1.3-1.7) | 0.011377 |
| Left middletemporal | 1.0 (0.4) | 1.0 (0.8-1.2) | 1.3 (0.3) | 1.3 (1.2-1.5) | 0.014345 |
| Left parahippocampal | 0.7 (0.2) | 0.6 (0.5-0.8) | 1.0 (0.2) | 0.9 (0.9-1.1) | 0.002717 |
| Left paracentral | 1.0 (0.3) | 0.9 (0.7-1.2) | 1.2 (0.3) | 1.3 (1.1-1.5) | 0.006416 |
| Left postcentral | 0.9 (0.3) | 0.7 (0.7-1.1) | 1.1 (0.3) | 1.2 (1.0-1.3) | 0.006871 |
| Left posteriorcingulate | 1.2 (0.4) | 1.2 (0.9-1.4) | 1.5 (0.3) | 1.5 (1.4-1.6) | 0.01056 |
| Left precentral | 0.9 (0.3) | 0.8 (0.7-1.0) | 1.1 (0.3) | 1.1 (0.9-1.2) | 0.01485 |
| Left rostralanteriorcingulate | 1.1 (0.3) | 1.1 (0.9-1.4) | 1.5 (0.3) | 1.5 (1.4-1.6) | 0.008233 |
| Left rostralmiddlefrontal | 1.3 (0.4) | 1.2 (1.0-1.5) | 1.6 (0.3) | 1.6 (1.4-1.8) | 0.019161 |
| Left superiorfrontal | 1.1 (0.4) | 1.0 (0.8-1.4) | 1.4 (0.3) | 1.5 (1.2-1.6) | 0.009931 |
| Left superiorparietal | 1.1 (0.4) | 1.0 (0.7-1.5) | 1.3 (0.3) | 1.3 (1.2-1.4) | 0.015865 |
| Left superiortemporal | 0.9 (0.3) | 0.9 (0.7-1.1) | 1.2 (0.3) | 1.1 (0.9-1.4) | 0.030355 |
| Left supramarginal | 1.1 (0.4) | 1.0 (0.8-1.4) | 1.3 (0.3) | 1.3 (1.2-1.4) | 0.031122 |
| Left insula | 0.9 (0.3) | 0.8 (0.7-1.0) | 1.1 (0.3) | 1.2 (0.9-1.3) | 0.013273 |
| Right caudalmiddlefrontal | 1.1 (0.3) | 1.1 (0.9-1.3) | 1.3 (0.3) | 1.4 (1.1-1.5) | 0.034899 |
| Right fusiform | 0.9 (0.3) | 0.9 (0.7-1.1) | 1.2 (0.3) | 1.1 (1.0-1.4) | 0.005922 |
| Right inferiorparietal | 1.1 (0.4) | 1.1 (0.8-1.3) | 1.3 (0.3) | 1.4 (1.3-1.5) | 0.016946 |
| Right inferiortemporal | 1.0 (0.3) | 1.0 (0.7-1.1) | 1.3 (0.3) | 1.3 (1.1-1.4) | 0.002504 |
| Right lingual | 0.7 (0.3) | 0.6 (0.5-0.7) | 0.8 (0.3) | 0.8 (0.7-0.9) | 0.007389 |
| Right parahippocampal | 0.7 (0.2) | 0.7 (0.6-0.9) | 1.0 (0.2) | 1.0 (0.9-1.0) | 0.001096 |
| Right paracentral | 1.0 (0.3) | 0.9 (0.8-1.2) | 1.2 (0.3) | 1.3 (0.9-1.4) | 0.023318 |
| Right postcentral | 0.9 (0.3) | 0.8 (0.6-1.1) | 1.1 (0.3) | 1.2 (1.0-1.2) | 0.033509 |
| Right posteriorcingulate | 1.2 (0.4) | 1.1 (0.9-1.4) | 1.4 (0.3) | 1.5 (1.3-1.6) | 0.036544 |
| Right precentral | 0.9 (0.3) | 0.8 (0.7-1.1) | 1.0 (0.3) | 1.1 (0.9-1.2) | 0.035522 |
| Right rostralanteriorcingulate | 1.2 (0.3) | 1.2 (0.9-1.4) | 1.4 (0.3) | 1.4 (1.3-1.6) | 0.042182 |
| Right rostralmiddlefrontal | 1.3 (0.4) | 1.3 (0.9-1.5) | 1.6 (0.3) | 1.7 (1.4-1.8) | 0.022121 |
| Right superiorfrontal | 1.1 (0.3) | 1.1 (0.8-1.3) | 1.4 (0.3) | 1.4 (1.2-1.6) | 0.022505 |
| Right superiorparietal | 1.0 (0.4) | 1.0 (0.7-1.2) | 1.3 (0.3) | 1.3 (1.1-1.4) | 0.015868 |
| Right superiortemporal | 0.9 (0.3) | 0.9 (0.6-1.1) | 1.1 (0.3) | 1.1 (0.9-1.3) | 0.034499 |
| Right insula | 0.9 (0.3) | 0.9 (0.7-1.0) | 1.1 (0.3) | 1.1 (0.9-1.3) | 0.016092 |

**B Tau (MCI)**

| **Region** | **Aff (-)** | | **Aff (+)** | | **p-value** |
| --- | --- | --- | --- | --- | --- |
|  | **Mean (SD)** | **Median (IQR)** | **Mean (SD)** | **Median (IQR)** |  |
| Left entorhinal | 1.7 (0.6) | 1.7 (1.2-2.1) | 2.0 (0.5) | 2.1 (1.8-2.2) | 0.035502 |
| Left isthmuscingulate | 1.4 (0.6) | 1.2 (1.1-1.5) | 1.4 (0.2) | 1.4 (1.2-1.6) | 0.047115 |
| Right isthmuscingulate | 1.4 (0.7) | 1.1 (1.0-1.4) | 1.4 (0.3) | 1.3 (1.2-1.5) | 0.049771 |
| Right parahippocampal | 1.4 (0.5) | 1.3 (1.1-1.6) | 1.5 (0.3) | 1.5 (1.4-1.6) | 0.033514 |

**C Aβ (AD)**

| **Region** | **Aff (-)** | | **Aff (+)** | | **p-value** |
| --- | --- | --- | --- | --- | --- |
|  | **Mean (SD)** | **Median (IQR)** | **Mean (SD)** | **Median (IQR)** |  |
| Left fusiform | 1.4 (0.2) | 1.4 (1.3-1.5) | 1.2 (0.3) | 1.1 (1.0-1.2) | 0.029813 |
| Left superiortemporal | 1.4 (0.2) | 1.5 (1.3-1.5) | 1.2 (0.3) | 1.3 (1.0-1.4) | 0.044337 |

**D Tau (AD)**

| **Region** | **Aff (-)** | | **Aff (+)** | | **p-value** |
| --- | --- | --- | --- | --- | --- |
|  | **Mean (SD)** | **Median (IQR)** | **Mean (SD)** | **Median (IQR)** |  |
| Right transversetemporal | 0.6 (0.4) | 0.7 (0.4-0.7) | 0.8 (0.2) | 0.9 (0.7-1.0) | 0.042601 |

**E Aβ and tau (MCI)**

| **Overlapping regions** |
| --- |
|  |
| Left isthmuscingulate |
| Right parahippocampal |

Supplementary Table 10. Regions with significantly different pathological accumulation between Aff (+) and Aff (-) (A) Aβ accumulation differences in MCI participants, (B) Tau accumulation differences in MCI participants, (C) Aβ accumulation differences in AD participants, (D) Tau accumulation differences in AD participants, (E) Brain regions showing differential Aβ and tau burden between Aff (+) and Aff (-) in MCI participants, chosen as seed regions for subsequent functional connectivity analysis. There were no overlapping regions in AD participants.

Abbreviations: Aff (-), group without affective factor; Aff (+), group with affective factor; MCI, mild cognitive impairment; AD, Alzheimer’s Disease; SD, standard deviation; IQR, interquartile range

**A Aβ (MCI)**

| **Region** | **Apa (-)** | | **Apa (+)** | | **p-value** |
| --- | --- | --- | --- | --- | --- |
|  | **Mean (SD)** | **Median (IQR)** | **Mean (SD)** | **Median (IQR)** |  |
| Left caudalmiddlefrontal | 1.1 (0.4) | 1.0 (0.8-1.4) | 1.4 (0.3) | 1.5 (1.4-1.5) | 0.013703 |
| Left cuneus | 0.8 (0.3) | 0.7 (0.6-0.9) | 1.0 (0.4) | 1.0 (0.7-1.2) | 0.033295 |
| Left entorhinal | 0.6 (0.2) | 0.6 (0.5-0.7) | 0.7 (0.2) | 0.7 (0.6-0.9) | 0.016645 |
| Left fusiform | 1.0 (0.4) | 0.9 (0.7-1.1) | 1.2 (0.2) | 1.2 (1.0-1.3) | 0.012084 |
| Left inferiorparietal | 1.1 (0.4) | 1.1 (0.8-1.4) | 1.4 (0.3) | 1.4 (1.2-1.6) | 0.031268 |
| Left inferiortemporal | 1.0 (0.4) | 1.0 (0.7-1.2) | 1.3 (0.2) | 1.3 (1.2-1.5) | 0.007704 |
| Left isthmuscingulate | 1.1 (0.3) | 1.1 (0.8-1.3) | 1.4 (0.2) | 1.4 (1.3-1.5) | 0.001242 |
| Left lingual | 0.7 (0.3) | 0.6 (0.4-0.7) | 0.8 (0.3) | 0.8 (0.6-0.9) | 0.021875 |
| Left medialorbitofrontal | 1.1 (0.4) | 1.1 (0.9-1.4) | 1.4 (0.3) | 1.4 (1.3-1.7) | 0.012036 |
| Left middletemporal | 1.0 (0.4) | 1.0 (0.8-1.2) | 1.4 (0.2) | 1.5 (1.2-1.5) | 0.010681 |
| Left parahippocampal | 0.7 (0.2) | 0.6 (0.5-0.9) | 1.0 (0.1) | 1.0 (0.9-1.1) | 0.000552 |
| Left paracentral | 1.0 (0.3) | 0.9 (0.7-1.1) | 1.3 (0.3) | 1.4 (1.3-1.5) | 0.001304 |
| Left parsopercularis | 1.1 (0.4) | 1.1 (0.8-1.3) | 1.4 (0.3) | 1.4 (1.4-1.6) | 0.012213 |
| Left parsorbitalis | 1.1 (0.3) | 1.1 (0.8-1.4) | 1.4 (0.2) | 1.4 (1.3-1.5) | 0.009349 |
| Left parstriangularis | 1.2 (0.4) | 1.2 (1.0-1.5) | 1.5 (0.3) | 1.5 (1.4-1.7) | 0.039279 |
| Left pericalcarine | 0.7 (0.6) | 0.6 (0.4-0.9) | 1.1 (0.6) | 0.9 (0.7-1.5) | 0.0333 |
| Left postcentral | 0.9 (0.3) | 0.7 (0.7-1.1) | 1.2 (0.2) | 1.3 (1.2-1.3) | 0.001714 |
| Left posteriorcingulate | 1.2 (0.4) | 1.2 (0.9-1.5) | 1.5 (0.2) | 1.5 (1.5-1.6) | 0.002228 |
| Left precentral | 0.9 (0.3) | 0.8 (0.7-1.0) | 1.1 (0.2) | 1.2 (1.1-1.3) | 0.003742 |
| Left precuneus | 1.2 (0.4) | 1.3 (1.0-1.5) | 1.6 (0.2) | 1.7 (1.5-1.7) | 0.000547 |
| Left rostralanteriorcingulate | 1.1 (0.3) | 1.1 (0.9-1.4) | 1.5 (0.3) | 1.5 (1.4-1.8) | 0.00994 |
| Left rostralmiddlefrontal | 1.3 (0.4) | 1.2 (1.0-1.5) | 1.6 (0.3) | 1.6 (1.5-1.7) | 0.029067 |
| Left superiorfrontal | 1.1 (0.4) | 1.0 (0.8-1.4) | 1.4 (0.3) | 1.5 (1.3-1.6) | 0.009458 |
| Left superiorparietal | 1.1 (0.4) | 1.0 (0.7-1.3) | 1.4 (0.2) | 1.5 (1.3-1.6) | 0.008728 |
| Left superiortemporal | 0.9 (0.3) | 0.9 (0.7-1.1) | 1.2 (0.2) | 1.3 (1.1-1.4) | 0.001565 |
| Left supramarginal | 1.1 (0.4) | 1.0 (0.8-1.3) | 1.4 (0.2) | 1.4 (1.3-1.5) | 0.007031 |
| Left transversetemporal | 0.9 (0.5) | 0.8 (0.6-1.1) | 1.4 (0.4) | 1.4 (1.2-1.6) | 0.003167 |
| Left insula | 0.8 (0.3) | 0.8 (0.7-1.0) | 1.2 (0.2) | 1.2 (1.1-1.3) | 0.001624 |
| Right caudalanteriorcingulate | 1.2 (0.3) | 1.1 (0.9-1.4) | 1.4 (0.2) | 1.4 (1.3-1.6) | 0.039434 |
| Right caudalmiddlefrontal | 1.1 (0.4) | 1.1 (0.8-1.3) | 1.4 (0.3) | 1.4 (1.2-1.5) | 0.041616 |
| Right inferiortemporal | 1.0 (0.3) | 1.0 (0.7-1.2) | 1.2 (0.2) | 1.2 (1.1-1.4) | 0.01608 |
| Right isthmuscingulate | 1.1 (0.3) | 1.1 (0.9-1.4) | 1.4 (0.2) | 1.3 (1.3-1.5) | 0.000971 |
| Right medialorbitofrontal | 1.1 (0.4) | 1.2 (0.8-1.3) | 1.4 (0.3) | 1.3 (1.3-1.6) | 0.010616 |
| Right middletemporal | 1.0 (0.4) | 1.0 (0.7-1.2) | 1.3 (0.2) | 1.3 (1.2-1.4) | 0.001678 |
| Right parahippocampal | 0.8 (0.2) | 0.7 (0.6-0.9) | 1.0 (0.2) | 1.0 (0.8-1.1) | 0.012844 |
| Right paracentral | 1.0 (0.3) | 0.9 (0.8-1.2) | 1.2 (0.3) | 1.4 (1.1-1.4) | 0.014987 |
| Right parsopercularis | 1.1 (0.4) | 1.2 (0.8-1.4) | 1.4 (0.2) | 1.3 (1.3-1.5) | 0.017087 |
| Right postcentral | 0.9 (0.3) | 0.8 (0.6-1.1) | 1.1 (0.2) | 1.2 (1.1-1.2) | 0.007131 |
| Right posteriorcingulate | 1.2 (0.4) | 1.2 (0.9-1.4) | 1.5 (0.3) | 1.5 (1.3-1.6) | 0.016452 |
| Right precentral | 0.9 (0.3) | 0.8 (0.7-1.1) | 1.1 (0.2) | 1.1 (1.0-1.2) | 0.028426 |
| Right precuneus | 1.2 (0.4) | 1.3 (0.9-1.5) | 1.5 (0.3) | 1.6 (1.3-1.7) | 0.016507 |
| Right rostralanteriorcingulate | 1.1 (0.3) | 1.2 (0.9-1.4) | 1.5 (0.3) | 1.4 (1.4-1.7) | 0.009031 |
| Right superiorfrontal | 1.1 (0.3) | 1.1 (0.8-1.3) | 1.4 (0.3) | 1.4 (1.2-1.6) | 0.011723 |
| Right superiortemporal | 0.9 (0.3) | 0.8 (0.6-1.0) | 1.2 (0.2) | 1.2 (1.1-1.4) | 0.003439 |
| Right supramarginal | 1.1 (0.3) | 1.0 (0.8-1.3) | 1.3 (0.2) | 1.3 (1.2-1.4) | 0.0049 |
| Right transversetemporal | 0.8 (0.5) | 0.7 (0.5-1.0) | 1.2 (0.4) | 1.3 (1.0-1.4) | 0.007131 |
| Right insula | 0.9 (0.3) | 0.9 (0.7-1.0) | 1.1 (0.2) | 1.1 (1.0-1.3) | 0.005597 |

**B Tau (MCI)**

| **Region** | **Apa (-)** | | **Apa (+)** | | **p-value** |
| --- | --- | --- | --- | --- | --- |
|  | **Mean (SD)** | **Median (IQR)** | **Mean (SD)** | **Median (IQR)** |  |
| Left isthmuscingulate | 1.4 (0.6) | 1.2 (1.1-1.4) | 1.5 (0.5) | 1.4 (1.2-1.7) | 0.040018 |
| Left middletemporal | 1.7 (0.8) | 1.4 (1.3-1.6) | 1.9 (0.5) | 1.8 (1.6-2.1) | 0.029354 |
| Left superiortemporal | 1.3 (0.5) | 1.2 (1.1-1.3) | 1.4 (0.2) | 1.5 (1.3-1.5) | 0.035431 |
| Right isthmuscingulate | 1.4 (0.6) | 1.1 (1.1-1.4) | 1.6 (0.6) | 1.4 (1.2-1.6) | 0.033295 |
| Right parahippocampal | 1.4 (0.5) | 1.3 (1.1-1.6) | 1.6 (0.4) | 1.5 (1.4-1.6) | 0.029354 |

**C Tau (AD)**

| **Region** | **Apa (-)** | | **Apa (+)** | | **p-value** |
| --- | --- | --- | --- | --- | --- |
|  | **Mean (SD)** | **Median (IQR)** | **Mean (SD)** | **Median (IQR)** |  |
| Left transversetemporal | 1.0 (0.5) | 0.9 (0.8-1.0) | 0.7 (0.3) | 0.7 (0.5-0.8) | 0.01802 |

**D Aβ and tau (MCI)**

| **Overlapping regions** |
| --- |
| Left isthmuscingulate |
| Left middletemporal |
| Left superiortemporal |
| Right isthmuscingulate |
| Right parahippocampal |

Supplementary Table 11. Regions with significantly different pathological accumulation between Apa (+) and Apa (-) (A) Aβ accumulation differences in MCI participants, (B) Tau accumulation differences in MCI participants, (C) Tau accumulation differences in AD participants, (D) Brain regions showing differential Aβ and tau burden between Apa (+) and Apa (-) in MCI participants, chosen as seed regions for subsequent functional connectivity analysis. There were no significantly differential Aβ accumulation and overlapping regions in AD participants.

Abbreviations: Apa (-), group without apathy factor; Apa (+), group with apathy factor; MCI, mild cognitive impairment; AD, Alzheimer’s Disease; SD, standard deviation; IQR, interquartile range

**A Aβ (MCI)**

| **Region** | **Hyp (-)** | | **Hyp (+)** | | **p-value** |
| --- | --- | --- | --- | --- | --- |
|  | **Mean (SD)** | **Median (IQR)** | **Mean (SD)** | **Median (IQR)** |  |
| Right caudalanteriorcingulate | 1.1 (0.3) | 1.1 (0.9-1.4) | 1.4 (0.3) | 1.4 (1.3-1.7) | 0.023387 |
| Right fusiform | 0.9 (0.4) | 0.9 (0.7-1.1) | 1.1 (0.2) | 1.1 (1.0-1.2) | 0.027191 |
| Right inferiorparietal | 1.1 (0.4) | 1.2 (0.8-1.4) | 1.3 (0.2) | 1.3 (1.2-1.4) | 0.048001 |
| Right inferiortemporal | 1.0 (0.3) | 1.0 (0.7-1.2) | 1.2 (0.2) | 1.2 (1.1-1.4) | 0.012126 |
| Right middletemporal | 1.0 (0.4) | 1.0 (0.7-1.2) | 1.3 (0.2) | 1.3 (1.2-1.4) | 0.006619 |
| Right parahippocampal | 0.8 (0.2) | 0.7 (0.6-0.9) | 0.9 (0.2) | 0.9 (0.7-1.1) | 0.039307 |
| Right postcentral | 0.9 (0.3) | 0.8 (0.6-1.1) | 1.1 (0.3) | 1.2 (0.8-1.3) | 0.024049 |
| Right precentral | 0.9 (0.3) | 0.8 (0.7-1.1) | 1.0 (0.2) | 1.1 (0.9-1.3) | 0.028885 |
| Right rostralanteriorcingulate | 1.1 (0.3) | 1.1 (0.9-1.4) | 1.5 (0.3) | 1.4 (1.4-1.6) | 0.006146 |
| Right superiorfrontal | 1.1 (0.3) | 1.1 (0.8-1.3) | 1.3 (0.3) | 1.4 (1.2-1.6) | 0.038262 |
| Right superiorparietal | 1.0 (0.4) | 1.0 (0.7-1.4) | 1.2 (0.2) | 1.2 (1.0-1.4) | 0.044591 |
| Right superiortemporal | 0.9 (0.3) | 0.8 (0.6-1.1) | 1.1 (0.2) | 1.1 (0.9-1.3) | 0.008237 |
| Right supramarginal | 1.1 (0.3) | 1.1 (0.8-1.3) | 1.3 (0.2) | 1.3 (1.1-1.5) | 0.007836 |
| Right transversetemporal | 0.8 (0.5) | 0.7 (0.5-1.0) | 1.1 (0.3) | 1.1 (0.9-1.3) | 0.005494 |
| Right insula | 0.9 (0.3) | 0.9 (0.7-1.0) | 1.1 (0.2) | 1.1 (1.1-1.2) | 0.011059 |

**B Aβ (AD)**

| **Region** | **Hyp (-)** | | **Hyp (+)** | | **p-value** |
| --- | --- | --- | --- | --- | --- |
|  | **Mean (SD)** | **Median (IQR)** | **Mean (SD)** | **Median (IQR)** |  |
| Left entorhinal | 0.6 (0.2) | 0.6 (0.5-0.7) | 0.8 (0.2) | 0.7 (0.6-0.9) | 0.040215 |
| Left lateraloccipital | 1.0 (0.1) | 1.0 (1.0-1.1) | 1.3 (0.2) | 1.3 (1.1-1.5) | 0.004308 |
| Right cuneus | 0.8 (0.1) | 0.8 (0.8-0.9) | 1.1 (0.3) | 1.1 (0.8-1.3) | 0.020122 |
| Right fusiform | 1.1 (0.2) | 1.1 (0.9-1.2) | 1.3 (0.2) | 1.4 (1.2-1.5) | 0.019403 |
| Right inferiortemporal | 1.3 (0.3) | 1.3 (1.1-1.4) | 1.5 (0.3) | 1.5 (1.4-1.7) | 0.042334 |
| Right lateraloccipital | 1.0 (0.1) | 1.1 (0.9-1.1) | 1.3 (0.3) | 1.3 (1.1-1.6) | 0.007032 |
| Right lingual | 0.7 (0.1) | 0.7 (0.7-0.8) | 1.0 (0.3) | 0.9 (0.7-1.2) | 0.036228 |
| Right pericalcarine | 0.9 (0.3) | 1.1 (0.6-1.2) | 1.4 (0.7) | 1.4 (1.0-1.8) | 0.04862 |
| Right postcentral | 1.0 (0.2) | 1.0 (1.0-1.2) | 1.3 (0.3) | 1.3 (1.2-1.5) | 0.014525 |
| Right supramarginal | 1.3 (0.2) | 1.3 (1.1-1.4) | 1.5 (0.3) | 1.4 (1.4-1.6) | 0.031016 |

**C Tau (AD)**

| **Region** | **Hyp (-)** | | **Hyp (+)** | | **p-value** |
| --- | --- | --- | --- | --- | --- |
|  | **Mean (SD)** | **Median (IQR)** | **Mean (SD)** | **Median (IQR)** |  |
| Left fusiform | 1.8 (0.6) | 1.6 (1.4-1.8) | 2.2 (0.5) | 2.2 (1.9-2.4) | 0.036275 |
| Left lateraloccipital | 1.7 (1.2) | 1.3 (1.1-1.6) | 1.9 (0.4) | 1.8 (1.6-2.1) | 0.018886 |
| Right lateraloccipital | 1.7 (0.9) | 1.2 (1.1-1.6) | 2.1 (0.8) | 2.0 (1.6-2.4) | 0.02238 |

**D Aβ and tau (AD)**

| **Region** |
| --- |
|  |
| Left lateraloccipital |
| Right lateraloccipital |

Supplementary Table 12. Regions with significantly different pathological accumulation between Hyp (+) and Hyp (-) (A) Aβ accumulation differences in MCI participants. There were no significantly differential tau accumulation and overlapping regions in MCI participants. (B) Aβ accumulation differences in AD participants, (C) Tau accumulation differences in AD participants, (D) Brain regions showing differential Aβ and tau burden between Hyp (+) and Hyp (-) in AD participants, chosen as seed regions for subsequent functional connectivity analysis.

Abbreviations: Hyp (-), group without hyperactivity factor; Hyp (+), group with hyperactivity factor; MCI, mild cognitive impairment; AD, Alzheimer’s Disease; SD, standard deviation; IQR, interquartile range

**A Tau (AD)**

| **Region** | **Psy (-)** | | **Psy (+)** | | **p-value** |
| --- | --- | --- | --- | --- | --- |
|  | **Mean (SD)** | **Median (IQR)** | **Mean (SD)** | **Median (IQR)** |  |
| Left postcentral | 1.2 (0.4) | 1.1 (1.0-1.1) | 1.0 (0.2) | 1.0 (0.9-1.0) | 0.046614 |
| Left precentral | 1.2 (0.5) | 1.0 (1.0-1.2) | 1.0 (0.1) | 0.9 (0.9-1.0) | 0.033789 |
| Right lateralorbitofrontal | 1.9 (0.8) | 1.5 (1.4-2.3) | 1.4 (0.2) | 1.4 (1.2-1.5) | 0.039783 |
| Right postcentral | 1.2 (0.2) | 1.1 (1.0-1.2) | 0.9 (0.1) | 0.9 (0.9-1.0) | 0.026567 |
| Right precentral | 1.3 (0.4) | 1.1 (1.0-1.4) | 1.0 (0.2) | 0.9 (0.8-1.1) | 0.024019 |
| Right superiorfrontal | 1.7 (0.9) | 1.2 (1.1-2.0) | 1.1 (0.2) | 1.1 (1.0-1.2) | 0.033789 |

Supplementary Table 13. Regions with significantly different pathological accumulation between Psy (+) and Psy (-) (A) Tau accumulation differences in AD participants. No significant differences in Aβ or tau accumulation were observed in MCI participants, and no significantly differences in Aβ accumulation were found in AD participants.

Abbreviations: Hyp (-), group without hyperactivity factor; Hyp (+), group with hyperactivity factor; MCI, mild cognitive impairment; AD, Alzheimer’s Disease; SD, standard deviation; IQR, interquartile range

| **Seed** | **Cluster** | **Cluster size (voxels)** | **Peak MNI coordinate  (x, y, z)** | **size  p-FWE** |
| --- | --- | --- | --- | --- |
| lICgG | Right frontal pole | 584 | +33 +43 +24 | 0.0098 |
|  | Right postcentral gyrus | 339 | +13 -39 +64 | 0.0403 |

**Supplementary Table 14. Significant clusters from comparisons of seed-to-voxel functional connectivity between groups with and without the affective factor in MCI participants** lICgG, left isthmus of cigntulate gyrus; FWE, family-wise error; MCI, mild cognitive impairment

| **Variables** | **β** | **Standard error** | **p-Value** |
| --- | --- | --- | --- |
| Intercept | 340.5336 | 212.9968 | 0.251 |
| Age | 0.4078 | 0.2281 | 0.216 |
| Sex | 2.4392 | 2.2944 | 0.399 |
| Education | -4.1518 | 2.6644 | 0.26 |
| MMSE | 2.1027 | 1.4024 | 0.273 |
| Connectivity between lICgG and right post cingulate gyrus | -2.6255 | 7.8618 | 0.77 |
| Connectivity between lICgG and right frontal pole | -7.4046 | 7.3478 | 0.42 |
| Tau SUVR in lICgG | -274.724 | 167.8899 | 0.243 |
| Amyloid SUVR in lICgG | -243.32 | 148.2043 | 0.242 |
| Tau : Amyloid SUVR in lICgG | 184.3198 | 112.2042 | 0.242 |

**Supplementary Table 15. Generalized linear model to predict the severity of the affective factor with lICgG in MCI group**

**Abbreviations:** MMSE, Mini-Mental State Examination; lICgG, left isthmus of cingulate gyrus; SUVR, standardized uptake value ratio; Tau : Amyloid SUVR, the interaction term of tau and amyloid SUVR; MCI, mild cognitive impairment

|  | NPS (-) | NPS (+) | Aff  (-) | Aff (+) | Apa  (-) | Apa (+) | Hyp (-) | Hyp (+) | Psy (-) | Psy (+) |
| --- | --- | --- | --- | --- | --- | --- | --- | --- | --- | --- |
| Medication | 5 (17.8) | 17  (37) | 9 (18.4) | 13 (52) | 12 (23.5) | 10  (43.5) | 13  (26) | 9  (37.5) | 16  (27.6) | 6  (37.5) |

**Supplementary Table 16. Proportion of participants taking anti-depressant and other behavioral medication by NPS group**

**Note:** Data are presented as number *(n)* with corresponding percentage *(%)*

**Abbreviations:** NPS (-), group without any neuropsychiatric symptomm; NPS (+), group with at least one NPS; Aff (-), group without the affective factor; Aff (+), group with the affective factor; Apa (-), group without the apathy factor; Apa (+), group with the apathy factor; Hyp (-), group without the hyperactivity factor; Hyp (+), group with the hyperactivity factor; Psy (-), group without the psychosis factor; Psy (+), group with the psychosis factor

**Supplementary Figure 1. Different functional connectivity of regions with accumulated amyloid-beta and tau between Aff (+) and Aff (-) in MCI participants (A)** Upper panel; A cluster in the postcentral gyrus (blue) and a cluster in the frontal pole (yellow) showed different FC with lICgG when contrasting Aff (+) from Aff (-) in MCI participants. The lICgG, a seed region, was selected among the regions with higher amyloid-beta and tau accumulation (Supplementary Table 10 A, B, E) based on the presence of significant functional connectivity differences. Lower panel; Distribution of individual functional connectivity strength between lICgG and the clusters.

Dots are z-transformed individual connectivity strength between the seed and the cluster.
