# Supplementary figures and images for: Neural Representations of Neuropsychiatric Symptoms in Alzheimer's Disease Continuum Using Pathology‐Based Functional Connectivity Analysis
Source: Brain Behav. 2025 Sep 1;15(9):e70774. doi: 10.1002/brb3.70774 (PMC12402403; doi:10.1002/brb3.70774)

A

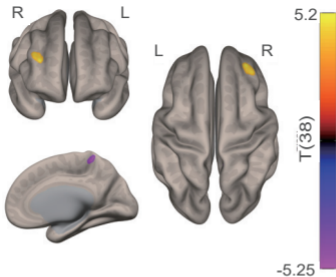

Seed : Left isthmus of cingulate gyrus (lICgG)

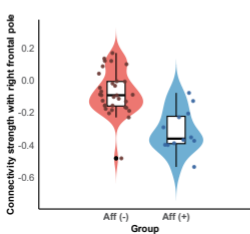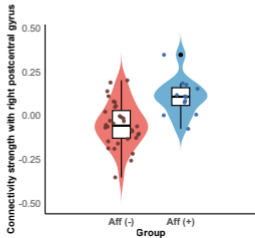

Supplement: Supplementary file 2 — Supporting figure S1: brb370774‐sup‐0002‐figureS1.pdf [file BRB3-15-e70774-s002.pdf]
